# Comparative efficacy and safety of attention-deficit/hyperactivity disorder pharmacotherapies, including guanfacine extended release: a mixed treatment comparison

**DOI:** 10.1007/s00787-017-0962-6

**Published:** 2017-03-03

**Authors:** Alain Joseph, Rajeev Ayyagari, Meng Xie, Sean Cai, Jipan Xie, Michael Huss, Vanja Sikirica

**Affiliations:** 1 Shire, Zug, Switzerland; 2 Analysis Group, Inc., Economic, Financial, and Strategy Consulting, 111 Huntington Avenue, 14th Floor, Boston, MA 02199 USA; 3 Analysis Group, Inc., New York, NY USA; 4 University Medicine, Department of Child and Adolescent Psychiatry, Mainz, Germany; 5 Shire, Wayne, PA USA

**Keywords:** Attention-deficit/hyperactivity disorder, Efficacy, Meta-analysis, Mixed treatment comparison, Children, Adolescents

## Abstract

This study compared the clinical efficacy and safety of attention-deficit/hyperactivity disorder (ADHD) pharmacotherapy in children and adolescents 6–17 years of age. A systematic literature review was conducted to identify randomized controlled trials (RCTs) of pharmacologic monotherapies among children and adolescents with ADHD. A Bayesian network meta-analysis was conducted to compare change in symptoms using the ADHD Rating Scale Version IV (ADHD-RS-IV), Clinical Global Impression–Improvement (CGI-I) response, all-cause discontinuation, and adverse event-related discontinuation. Thirty-six RCTs were included in the analysis. The mean (95% credible interval [CrI]) ADHD-RS-IV total score change from baseline (active minus placebo) was −14.98 (−17.14, −12.80) for lisdexamfetamine dimesylate (LDX), −9.33 (−11.63, −7.04) for methylphenidate (MPH) extended release, −8.68 (−10.63, −6.72) for guanfacine extended release (GXR), and −6.88 (−8.22, −5.49) for atomoxetine (ATX); data were unavailable for MPH immediate release. The relative risk (95% CrI) for CGI-I response (active versus placebo) was 2.56 (2.21, 2.91) for LDX, 2.13 (1.70, 2.54) for MPH extended release, 1.94 (1.59, 2.29) for GXR, 1.77 (1.31, 2.26) for ATX, and 1.62 (1.05, 2.17) for MPH immediate release. Among non-stimulant pharmacotherapies, GXR was more effective than ATX when comparing ADHD-RS-IV total score change (with a posterior probability of 93.91%) and CGI-I response (posterior probability 76.13%). This study found that LDX had greater efficacy than GXR, ATX, and MPH in the treatment of children and adolescents with ADHD. GXR had a high posterior probability of being more efficacious than ATX, although their CrIs overlapped.

**Electronic supplementary material:**

The online version of this article (doi:10.1007/s00787-017-0962-6) contains supplementary material, which is available to authorized users.

## Background

The current UK National Institute for Health and Care Excellence (NICE) guidelines for attention-deficit/hyperactivity disorder (ADHD) recommend medication for patients with severe impairment or for those patients with moderate impairment who have refused non-drug treatments or whose symptoms have not responded to other, non-drug interventions [1]. Monotherapy with stimulants, including amphetamines and methylphenidate (MPH), is the mainstay of pharmacologic treatment for ADHD in children and adolescents; however, about 20–35% of patients are refractory or intolerant to stimulants [2–5]. In these cases, alternative options include non-stimulants and behavioral therapies. These treatments may be in the form of monotherapy or, in case of refractoriness or intolerance to monotherapy, adjunctive therapy (e.g., add-on therapy) to existing stimulants to help improve symptoms that cannot be controlled by stimulants alone.

European guidelines for the management of ADHD in children and adolescents [6] include monotherapy with stimulants, such as dexamphetamine (d-AMPH), lisdexamfetamine dimesylate (LDX), and MPH, either immediate release (IR) or extended release (ER)/osmotic-release oral system (OROS). For those children and adolescents refractory or intolerant to stimulants, alternative options include non-stimulants, such as atomoxetine (ATX), clonidine immediate release (CIR), and guanfacine immediate release (GIR). The NICE guidelines [1] recommend ATX and MPH for the management of ADHD in children and adolescents aged 6–17 years. Guanfacine extended release (GXR) is a non-stimulant approved for both monotherapy and adjunctive therapy in children and adolescents (6–17 years) in the USA and Canada. In addition, GXR was approved by the European Medicines Agency (EMA) for children and adolescents (6–17 years) in September 2015. The availability of treatments with different mechanisms of action provides opportunities for additional treatment strategies when patients do not adequately respond to prior therapies.

The objective of this study was to compare the clinical efficacy and safety of ADHD pharmacotherapies in children and adolescents aged 6–17 years. A number of randomized clinical trials have compared the efficacy and safety of these pharmacotherapies with each other or with placebo in children and adolescents. A recently published study conducted a meta-analysis incorporating published data from randomized controlled trials (RCTs) containing GXR and placebo [7]. However, the present study has a broader scope, performing a mixed treatment comparison (MTC) of EMA-approved pharmacotherapies and GXR in a network meta-analysis framework. It incorporates both direct and indirect evidence, from a wider range of sources.

## Methods

### Search strategy and inclusion/exclusion criteria

The review included RCTs with publications reporting the safety and efficacy of pharmacotherapies for the treatment of ADHD in children and adolescents. The following electronic databases were included in the search: MEDLINE; EMBASE; PsychINFO; Cochrane Central Register of Controlled Trials; CINAHL; and Science Citation Index. The last 4 years of the following conferences were also included: European Congress of Psychiatry (European Psychiatric Association; EPA); and European Society of Child and Adolescent Psychiatry (ESCAP) Congress. Abstracts from the American Academy of Child and Adolescent Psychiatry (AACAP) annual meeting were included for 2012–2014; 2015 abstracts could not be accessed.

The search included articles indexed through May 2016. No time restriction was applied to database searches of the drugs. Detailed search criteria are available in Online Resource 1.

Inclusion and exclusion criteria were designed to broadly identify available information on treatments for ADHD in children and adolescents. Studies were eligible if they satisfied the following conditions:

- A placebo or active comparator RCT.
- Included children or adolescents aged 6–17 years with ADHD.
- Interventions included monotherapy with at least one drug of interest (i.e., d-AMPH, ATX, CIR, GIR, GXR, LDX, MPH-IR, or MPH-ER/OROS).
- Reported efficacy or safety outcomes after a treatment duration of ≤16 weeks.
- Included ˃25 patients.

Trials were excluded if all patients had a comorbid condition in addition to ADHD. In addition, as the comparison with non-stimulants was a novel element of this study, trials were excluded if they could not be connected to GXR and ATX in an evidence network via a chain of common comparators. As a consequence, only parallel-arm studies were included—crossover studies were excluded if they did not report results for the parallel phase (just prior to crossover).

RCTs identified in the initial search were screened for consistency with the inclusion and exclusion criteria. Data extraction was then performed on the selected studies. Means and standard errors for continuous outcomes and rates for dichotomous outcomes were extracted from the publications for the included trials. In addition, patient baseline characteristics and characteristics of the randomized dosing arms, including sample size, age, sex, race, baseline ADHD Rating Scale Version IV (ADHD-RS-IV) total score, and comorbidities, were extracted.

### Outcomes

The following efficacy outcomes were assessed for possible inclusion in meta-analyses:

- Change from baseline-to-study endpoint in ADHD-RS-IV total score. ADHD-RS-IV is a symptom measurement scale where the total score ranges from 0 to 54, with a higher score indicating more severe ADHD [8].
- Achievement of Clinical Global Impression–Improvement (CGI-I) response. CGI-I measures overall improvement in ADHD. CGI-I response was defined as a CGI-I score of 1 (very much improved) or 2 (much improved) [9].
- Clinical Global Impression–Severity (CGI-S). CGI-S measures severity of ADHD on a scale from 1 to 7 [9].
- Conners’ Parent Rating Scales (CPRSs). The CPRSs consist of items scored by parents on a 4-point scale. The short form comprises 27 items, while the long form comprises 80 items [10].
- Swanson, Nolan, and Pelham Scale (SNAP-IV). SNAP-IV is a 90-item rating scale, with each item rated on a 4-point scale [11].

Safety outcomes considered were all-cause discontinuation and discontinuation due to adverse events (AEs). The feasibility of forming a connected network for each outcome was assessed based on data availability in the systematic literature review results. Different outcomes were not combined in any way in the analyses.

### Quality assessment

The quality of studies was assessed according to the following criteria from the NICE specification for manufacturer/sponsor submission of evidence in single technology appraisals [12]:

- Randomization was carried out appropriately.
- Concealment of treatment allocation was adequate.
- Groups were similar at the outset of the study in terms of prognostic factors.
- Care providers, participants, and outcome assessors were blind to treatment allocation.
- Study was assessed for any unexpected imbalances in dropouts between groups.
- Study was assessed for evidence suggestive that the authors measured more outcomes than they reported.
- Study was assessed for whether the analysis included an intention-to-treat analysis, whether this method was appropriate, and, if so, and whether appropriate methods were used to account for missing data.

Two reviewers independently rated the screening, data extraction, and quality of each study, and discrepancies between the reviewers were resolved through consultation with a third reviewer.

### Core and sensitivity mixed treatment comparisons

Each outcome was assessed in a core analysis and a set of sensitivity analyses. The specific analyses performed were dependent on the data available from the systematic literature review.

#### Core analyses

The core analysis for each identified outcome had the following characteristics:

- A random-effects model for the effect of each treatment compared with placebo, with one treatment arm per drug, and including study-level random effects to account for heterogeneity in the outcome.
- Adjusted for baseline covariates to the extent that data were available.
- Excluded short-term studies (≤3 weeks).

When conducting the MTC, all doses of a particular drug were treated as the same. For example, study arms receiving GXR at 2, 3, or 4 mg/day would all be considered as the same treatment, GXR.

#### Sensitivity analyses

Sensitivity analyses were performed for each outcome to assess the robustness of the findings to variations in the methodology. Adjustments for different sets of baseline covariates were considered. Variations in the statistical method used to account for outcome heterogeneity (fixed versus random effects) were explored. In addition, short-term studies (duration ≤3 weeks) were also considered for inclusion in a sensitivity analysis if this substantially increased the number of trials that could be included in the analysis.

### Statistical methodology

Indirect comparison methods can be used when there is interest in comparing treatments for which no head-to-head trials exist. MTCs are a special case that allow the simultaneous estimation of the effects of multiple treatments and can incorporate both indirect and direct comparisons [13]. The MTC methodology was chosen for the statistical analysis in order to simultaneously evaluate the effects of these treatments and because there were both indirect and direct comparisons between the treatments of interest. In accordance with the most recent guidance from the NICE Decision Support Unit technical support documents and ISPOR good practices for indirect comparison, a Bayesian network meta-analysis approach to MTC was used [14, 15]. Bayesian network meta-analysis models for MTCs were fit to estimate the effect of each treatment on each feasible efficacy or safety outcome. The analyses were implemented using the statistical software R (https://cran.r-project.org). Gibbs sampling for Bayesian inference was performed using JAGS (http://mcmc-jags.sourceforge.net).

An estimate of treatment effect comparing each treatment with a common comparator (i.e., placebo) was generated using the network meta-analysis. For continuous outcomes, a normal likelihood model with linear link was employed, and the difference from placebo in mean baseline-to-endpoint change in score was reported. For categorical outcomes, a binomial likelihood model with logit link was employed, and relative rates were estimated using a pooled placebo rate. The placebo rate was computed by pooling all the placebo arms in the data, weighted by sample size. Specifically, this was done by first generating placebo-arm rates using their sampling distributions, and incorporating the placebo-arm risk into the Bayesian model in accordance with NICE guidelines.

The core analyses incorporated a meta-regression adjustment for differences in baseline characteristics among the included studies (Online Resource 2). The baseline characteristics included in the meta-regression were those that were consistently measured and available in the included trials in each analysis. Additional meta-regression adjustments for covariates were included as sensitivity analyses where possible, including models with no meta-regression adjustment, as well as models that included additional covariates in the adjustment at the cost of a reduction in the number of trials that could be included in the analysis.

Prior information about the treatment effects, which was needed for the Bayesian analyses, was chosen to be non-informative in order to avoid a preference for any specific values of the treatment effects and to avoid biasing the parameter estimates. Markov chain Monte Carlo methods were used to obtain posterior distributions in the Bayesian analysis. A Gibbs sampler with 50,000 burn-in iterations and an additional 50,000 iterations was implemented to generate the posterior distributions for the treatment effects for each outcome of interest. Convergence of the Gibbs sampler was assessed using the Geweke method and Heidelberg–Welch stationarity and half-width tests [16–19]. The uncertainty of the estimates of treatment effects were summarized using 95% Bayesian credible intervals (CrIs) based on the 2.5 and 97.5 percentiles of the posterior distribution of the treatment effects. Non-overlap of 95% CrIs was interpreted as strong evidence that the treatment effects differ. In addition, the probability that each drug had the highest efficacy among all treatments was estimated based on posterior distributions. GXR was also compared individually with each of the other drugs, and the efficacy and discontinuation probability rates in comparison with each of the other drugs were reported. In addition, posterior density plots of the posterior distributions of the treatment effects were produced for each of the analyses.

Heterogeneity in the evidence network for the efficacy analyses was assessed using methods suggested in the NICE guidelines on evidence synthesis. Both fixed- and random-effects models were fitted to account for different assumptions regarding heterogeneity of treatment effects. Fixed-effects models assume a common treatment effect for a given treatment across studies; random-effects models assume that the treatment effects are not necessarily the same but are exchangeable in the sense that, while the actual treatment effect may vary across trials by chance, there are no structural differences between the trials. As a primary assessment of heterogeneity, the deviance information criterion (DIC) was compared between the fixed- and random-effects models. In addition, based on NICE guidelines [20], outcome heterogeneity was assessed (without regard to the meta-regression adjustments used in the analysis) using *I* ^2^ and Cochran’s *Q* measures in trials comparing all pairs of drugs that were compared in two or more trials. Use of these three heterogeneity measures is consistent with NICE recommendations [20].

## Results

### Systematic literature review

#### Search and screening

A total of 10,442 records, including database searches and conferences satisfied the initial search criteria. After an assessment of data availability following screening, three outcomes (CGI-S, CPRS, and SNAP-IV) were dropped from the analyses due to the absence of sufficiently many studies reporting these outcomes to form connected networks to compare these outcomes through common comparators. Sufficient data were available to form a network of treatments for the following outcomes: change in ADHD-RS-IV total score, CGI-I response, all-cause discontinuation, and discontinuation due to AEs. No data on MPH-IR were available for change in ADHD-RS-IV total score.

Data availability allowed for comparison of GXR, LDX, ATX, MPH-ER/MPH-OROS, and MPH-IR. The treatments d-AMPH, CIR, and GIR could not be included in any of the networks due to insufficient data to populate the network. Not all trials with MPH identified the intervention as extended or immediate release. Hence, an additional classification step was undertaken whereby MPH treatment was classified as MPH-IR if the drug was administered more than twice a day, and as MPH-ER otherwise. This classification was further reviewed to determine whether the classification was reasonable, and the impact on the findings was assessed by considering the number and size of the studies for which the classification was necessary. Of 20 RCTs that included an MPH arm, 4 articles did not specify whether the MPH formulation was ER or IR. Of these 4 articles, 3 were small (with MPH sample sizes of 20 [24], 32 [27], and 90 [59]) and one was moderate sized (MPH sample size of 166 [56]). Details are provided in Online Resource 2. A sensitivity analysis was performed, changing the classification for the largest study from MPH-ER to MPH-IR. All MPH-OROS studies were classified as MPH-ER.

**Fig. 1:**
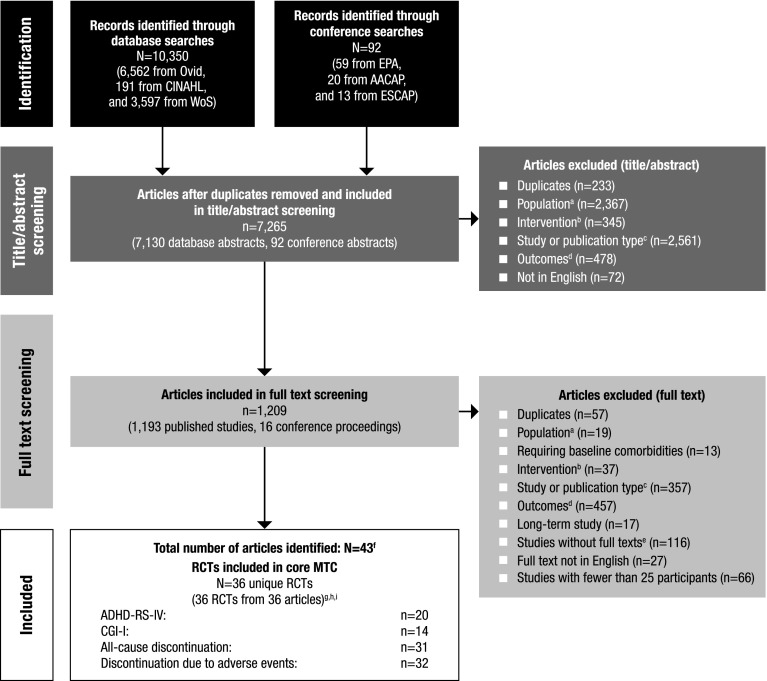
PRISMA diagram for the efficacy and safety of ADHD treatments for children and adolescents. ^a^Exclusions for non-human studies, not an ADHD population, and studies focusing on adults only (i.e., no children or adolescents included). ^b^Did not include treatments of interest (methylphenidate, atomoxetine, dexamphetamine, lisdexamfetamine/lisdexamphetamine, clonidine, or guanfacine). ^c^Preclinical studies, Phase I studies, prognostic studies, retrospective studies, case reports, audio files, commentaries and letters (publication type), consensus reports, nonsystematic reviews, or titles without corresponding abstracts (title/abstract screening only). ^d^For title/abstract screening, studies reporting only pharmacokinetics or biochemical, neuroimaging, or genetic outcomes were excluded. For full text screening, studies that did not report at least one of the following efficacy measures commonly used in child and adolescent ADHD were excluded: ADHD-RS-IV, CGI scales, Conners’ Parent Rating Scale, or Swanson, Nolan, and Pelham scale. All studies of clonidine IR and guanfacine IR were excluded at this stage because none of the efficacy outcomes reported in those studies could be used to form a connected network that included guanfacine extended release and atomoxetine. ^e^Including studies for which the full text is not available for open access download or for purchase. ^f^Six short-term studies were included in the sensitivity analyses of CGI-I comparison: Wilens et al. [21], Kemner et al. [22], Greenhill et al. [23], Pliszka et al. [24], Biederman et al. [25], and Findling et al. [26]. Additionally, one study not reporting baseline characteristics was included in the sensitivity analyses of all-cause discontinuation: Ialongo et al. [27]. ^g^When full or precise information was not available in the publication (e.g., results shown in a graph, results for pooled treatment arms, standard errors not reported) and the CSR was available, numbers were extracted from the CSR. CSRs were used to extract information on ADHD-RS-IV mean change and standard errors corresponding to Biederman et al. [28, 29] and Sallee et al. [30]. Two studies that did not report standard errors for ADHD-RS-IV were excluded. ^h^One publication contained two studies [31]. We considered these to be two separate RCTs, and labeled them as Spencer et al. [31]. However, we identified an additional publication of one of these studies, which is included in the count of articles. ^i^Due to differences in the outcomes reported in the RCTs, a total of 36 distinct RCTs were included in the four mixed treatment comparison analyses, although no more than 32 RCTs reported any one outcome. *AACAP* American Academy of Child and Adolescent Psychiatry, *ADHD* attention-deficit/hyperactivity disorder, *ADHD-RS-IV* Attention-Deficit/Hyperactivity Disorder Rating Scale Version IV, *CGI-I* Clinical Global Impression–Improvement, *CSR* clinical study report, *EPA* European Psychiatric Association, *ESCAP* European Society of Child and Adolescent Psychiatry, *IR* immediate release, *MTC* mixed treatment comparison, *RCT* randomized controlled trial

**Fig. 2:**
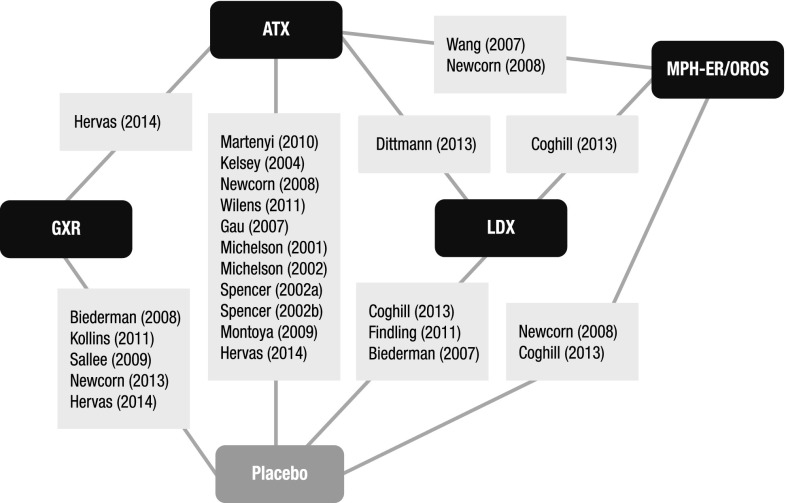
Evidence network for ADHD-RS-IV total score change from baseline. Methylphenidate immediate release, clonidine immediate release, and guanfacine immediate release could not be included in the network due to lack of data. Trials: Biederman [28]; Biederman [29]; Coghill [33]; Dittmann [35]; Findling [37]; Gau [40]; Hervas [41]; Kelsey [42]; Kollins [43]; Martenyi [45]; Michelson [46]; Michelson [47]; Montoya [48]; Newcorn [49]; Newcorn [50]; Sallee [30]; Spencer [31]; Wang [56]; Wilens [60]. *ADHD-RS-IV* Attention-Deficit/Hyperactivity Disorder Rating Scale Version IV, *ATX* atomoxetine, *GXR* guanfacine extended release, *LDX* lisdexamfetamine dimesylate, *MPH-OROS/ER* methylphenidate extended release/osmotic-release oral system

**Fig. 3:**
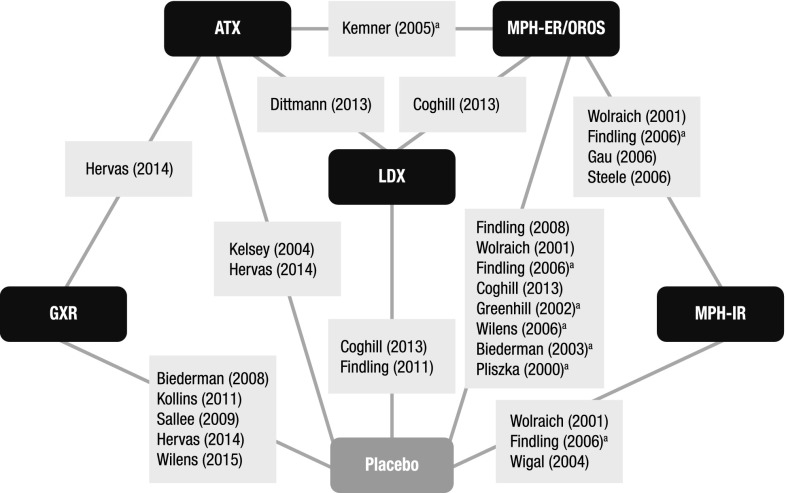
Evidence network for CGI-I response, defined as a CGI-I score of 1 or 2. Clonidine immediate release and guanfacine immediate release could not be included in the network due to lack of data. ^a^Indicates short-term studies, which are not included in the core analysis but are included in sensitivity analysis. Trials: Biederman [25]; Biederman [29]; Coghill [33]; Dittmann [35]; Findling [26]; Findling [36]; Findling [37]; Gau [39]; Greenhill [23]; Hervas [41]; Kelsey [42]; Kemner [22]; Kollins [43]; Pliszka [24]; Sallee [30]; Steele [53]; Wigal [59]; Wilens [21]; Wilens [61]; Wolraich [62]. *ATX* atomoxetine, Clinical Global Impression–Improvement, *GXR* guanfacine extended release, *LDX* lisdexamfetamine dimesylate, *MPH-OROS/ER* methylphenidate extended release/osmotic-release oral system, *MPH-IR* methylphenidate immediate release

**Fig. 4:**
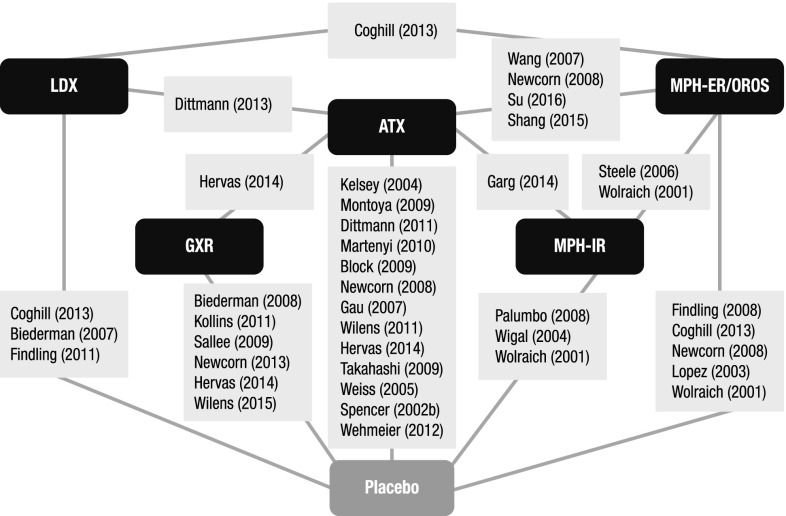
Evidence network for all-cause discontinuation Clonidine immediate release and guanfacine immediate release could not be included in the network due to lack of data. ^a^Study does not report baseline age or percent female and thus was not included in the core analysis but was included in the sensitivity analyses. Trials: Biederman [28]; Biederman [29]; Block [32]; Coghill [33]; Dittmann [34]; Dittmann [35]; Findling [36]; Findling [37]; Garg [38]; Gau [40]; Hervas [41]; Ialongo [27]; Kelsey [42]; Kollins [43]; López [44]; Martenyi [45]; Montoya [48]; Newcorn [49]; Newcorn [50]; Palumbo [51]; Sallee [30]; Shang [52]; Spencer [31]; Steele [53]; Su [54]; Takahashi [55]; Wang [56]; Weiss [58]; Wilens [60]; Wilens [61]; Wolraich [62]. *ATX* atomoxetine, *GXR* guanfacine extended release, *LDX* lisdexamfetamine dimesylate, *MPH-ER/OROS* methylphenidate extended release/osmotic-release oral system, *MPH-IR* methylphenidate immediate release

**Fig. 5:**
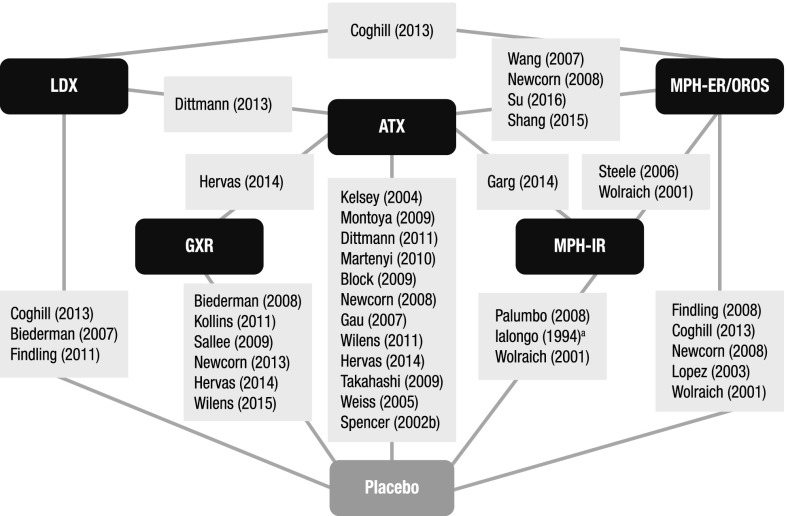
Evidence network for discontinuation due to adverse events. Clonidine immediate release and guanfacine immediate release could not be included in the network due to lack of data. Trials: Biederman [28]; Biederman [29]; Block [32]; Coghill [33]; Dittmann [34]; Dittmann [35]; Findling [36]; Findling [37]; Garg [38]; Gau [39]; Hervas [41]; Kelsey [42]; Kollins [43]; López [44]; Martenyi [45]; Montoya [48]; Newcorn [49]; Newcorn [50]; Palumbo [51]; Sallee [30]; Shang [52]; Spencer [31]; Steele [53]; Su [54]; Takahashi [55]; Wang [56]; Wehmeier [57]; Weiss [58]; Wigal [59]; Wilens [60]; Wilens [61]; Wolraich [62]. *ATX* atomoxetine; *GXR* guanfacine extended release, *LDX* lisdexamfetamine dimesylate, *MPH-ER/OROS* methylphenidate extended release/osmotic-release oral system, *MPH-IR* methylphenidate immediate release

A total of 36 RCTs met the screening criteria and were included in the analysis for at least one of the outcomes (Fig. 1; Table 1). In total, 20 articles were included in the network for ADHD-RS-IV total score change (Fig. 2), 14 for CGI-I response (Fig. 3), 31 for all-cause discontinuation (Fig. 4), and 32 for discontinuation due to AEs (Fig. 5). All of the trial results were published between 1994 and 2016. Of the 36 RCTs, 6 trials had GXR arms, 4 trials had LDX arms, 21 trials had ATX arms, 10 trials had MPH-ER arms, and 6 trials had MPH-IR arms. Among these, 1 trial had GXR and ATX arms, 1 trial had LDX and ATX arms, 1 trial had LDX and MPH-ER arms, 4 trials had ATX and MPH-ER arms, 1 trial had ATX and MPH-IR arms, and 3 trials had MPH-ER and MPH-IR arms. The same 36 RCTs were also used in the majority of sensitivity analyses. In addition, six short-term studies (duration ≤3 weeks) were included in sensitivity analyses of CGI-I comparison: Biederman et al. [25], Findling et al. [26], Greenhill et al. [23], Kemner et al. [22], Pliszka et al. [24], and Wilens et al. [21]; one study (Ialongo et al. [27]) not reporting baseline percent female or age was included in the sensitivity analyses of all-cause discontinuation.

**Table 1:** Study outcomes by trial

Article	Included in ADHD-RS-IV change	Included in CGI-I response	Included in all-cause discontinuation	Included in AE discontinuation	Reports mean age	Reports mean percent female	Reports mean baseline ADHD-RS-IV score	Short-term study (≤3 weeks)
Biederman [25]		X			✓	✓		Short-term
Biederman [28]	X		X	X	✓	✓	✓	
Biederman [29]	X	X	X	X	✓	✓	✓	
Block [32]			X	X	✓	✓		
Coghill [33]	X	X	X	X	✓	✓	✓	
Dittmann [34]			X	X	✓	✓		
Dittmann [35]	X	X	X	X	✓	✓	✓	
Findling [26]		X			✓	✓		Short-term
Findling [36]		X	X	X	✓	✓	✓	
Findling [37]	X	X	X	X	✓	✓	✓	
Garg [38]			X	X	✓	✓		
Gau [39]		X			✓	✓		
Gau [40]	X		X	X	✓	✓	✓	
Greenhill [23]		X			✓	✓		Short-term
Hervas [41]	X	X	X	X	✓	✓	✓	
Ialongo [27]			X					
Kelsey [42]	X	X	X	X	✓	✓	✓	
Kemner [22]		X			✓	✓	✓	Short-term
Kollins [43]	X	X	X	X	✓	✓		
López [44]			X	X	✓	✓		
Martenyi [45]	X		X	X	✓	✓	✓	
Michelson [46]	X				✓	✓	✓	
Michelson [47]	X				✓	✓	✓	
Montoya [48]	X		X	X	✓	✓	✓	
Newcorn [49]	X		X	X	✓	✓	✓	
Newcorn [50]	X		X	X	✓	✓	✓	
Palumbo [51]			X	X	✓	✓		
Pliszka [24]		X			✓			Short-term
Sallee [30]	X	X	X	X	✓	✓	✓	
Shang [52]			X	X	✓	✓		
Spencer [31]	X				✓	✓	✓	
Spencer [31]	X		X	X	✓	✓	✓	
Steele [53]		X	X	X	✓	✓		
Su [54]			X	X	✓	✓		
Takahashi [55]			X	X	✓	✓		
Wang [56]	X		X	X	✓	✓	✓	
Wehmeier [57]				X	✓	✓	✓	
Weiss [58]			X	X	✓	✓		
Wigal [59]		X		X	✓	✓		
Wilens [21]		X			✓	✓	✓	Short-term
Wilens [60]	X		X	X	✓	✓	✓	
Wilens [61]		X	X	X	✓	✓		
Wolraich [62]		X	X	X	✓	✓		

Baseline covariates were chosen for availability, consistency across studies, and potential clinical significance. Mean covariate values were weighted by the treatment-arm sample sizes for each outcome

*ADHD-RS-IV* Attention-Deficit/Hyperactivity Disorder Rating Scale Version IV, *AE* adverse event, *CGI-I* Clinical Global Impression–Improvement

#### RCT characteristics

All of the studies used the DSM-IV criteria, except for the study of Pliszka et al. [24], which used the diagnostic interview schedule for children [63] and the study of Ialongo et al. [27], which used the DSM-II criteria. Study durations ranged from 3 to 16 weeks for the double-blind period. Sample sizes ranged from 16 to 222 subjects per arm; the total number of patients ranged from 1987 for ATX in the AE-related discontinuation analysis to 230 for MPH-IR in the all-cause discontinuation analysis (Table 2). Mean age at baseline ranged from 8.5 to 14.6 years and the proportion of females ranged from 9.4 to 36.1%. The mean baseline ADHD-RS-IV total score was available in 22 studies and ranged from 31.5 to 43.5. Across the analyses of various outcomes, the average dose ranged from 2.87 to 4.30 mg/day for GXR, 44.40 to 51.52 mg/day for LDX, 17.46 to 47.09 mg/day for ATX, 18.00 to 39.03 mg/day for MPH-ER, and 17.35 to 30.76 mg/day for MPH-IR.

**Table 2:** Sample sizes for the core analyses

	ADHD-RS-IV total score change	CGI-I response	All-cause discontinuation	AE discontinuation
Placebo	1262	930	1675	1779
ATX	1465	370	1924	1987
GXR	958	894	1130	1130
LDX	673	453	697	697
MPH-ER	491	394	1062	1062
MPH-IR	0	245	230	276

*ADHD-RS-IV* Attention-Deficit/Hyperactivity Disorder Rating Scale Version IV, *AE* adverse event, *ATX* atomoxetine, *CGI-I* Clinical Global Impression–Improvement, *GXR* guanfacine extended release, *MPH-ER* methylphenidate extended release, *MPH-IR* methylphenidate immediate release, *LDX* lisdexamfetamine dimesylate

Quality assessment criteria were examined for all included studies (Table 3). All of the studies were blinded, except for Kemner et al. [22], Steele et al. [53], Gau et al. [39], Garg et al. [38], Shang et al. [52], and Su et al. [54], which were open-label studies. The method of ADHD-RS-IV rating was generally consistent across trials. The most common scoring method was the ADHD-RS-IV Parent Version: Investigator Administered and Scored. All except two studies reporting ADHD-RS-IV specified that the measure was investigator scored, with most further specifying that the scoring was based on interviews with parents. Dittman et al. [35] and Hervas et al. [41] were not specific about scoring, but did not indicate that any other scoring method was used.

**Table 3:** Quality assessment of included studies

Article	Randomization carried out appropriately?	Concealment of treatment allocation adequate?	Groups similar at the outset of the study in terms of prognostic factors?	Care providers, participants, and outcome assessors blind to treatment allocation?	Unexpected imbalances in dropouts between groups?	Evidence suggestive that the authors measured more outcomes than they reported?	Did the analysis include an intention-to-treat analysis? If so, was this appropriate and were appropriate methods used to account for missing data?
Biederman [25]	Yes	Yes	Yes	Unclear	Unclear	No	Yes and appears appropriate, LOCF was used for missing data
Biederman [28]	Yes	Yes	Yes	Yes	No	No	Yes and appears appropriate, methods used for missing data not reported
Biederman [29]	Presumably yes	Yes	Yes	Yes	No	No	Yes and appears appropriate, methods used for missing data not reported
Block [32]	Yes	Yes	Yes	Yes	No	No	Yes and appears appropriate, LOCF was used for missing data
Coghill [33]	Yes (stratified by country, age group)	Yes	Yes	Yes	Yes: notably more dropouts for PBO (around 2 × as many as for LDX or MPH-OROS)	No	Not mentioned
Dittmann [34]	Yes (stratified by age)	Yes	Yes	Unclear	Yes (PBO: 37.3%, ATX-fast: 26.7%, ATX-slow: 21.3%)	No	Yes and appears appropriate, LOCF was used for missing data
Dittmann [35]	Yes	Yes	Yes	Yes	No	No	Yes, efficacy data were analyzed using the full analysis set, LOCF was used for missing data
Findling [26]	Yes	Unclear	Yes	Unclear	No	No	Yes and appears appropriate, LOCF was used for missing data
Findling [36]	Yes	Yes	Yes	Yes	No	No	Yes and appears appropriate, methods used for missing data not reported
Findling [37]	Yes	Yes	Yes	Yes	No	No	Yes and appears appropriate, methods used for missing data not reported
Garg [38]	Yes	No: open label	No: 3% of MPH-IR patients had baseline comorbidities compared to 16.7% of ATX patients	No: open label	Yes: Discontinuation rates 18.2% (MPH-IR) versus 30.6% (ATX)	Yes: blood pressure noted as a secondary outcome in methods, but not reported in results	Not mentioned
Gau [39]	Unclear	No: open label	Yes	No: open label	No	No	Yes and appears appropriate, methods used for missing data not reported
Gau [40]	Yes	Yes	Yes	Yes	No	No	Yes and appears appropriate, LOCF was used for missing dat.
Greenhill [23]	Yes	Unclear	Yes	Yes	Yes	No	Yes and appears appropriate, LOCF was used for missing data
Hervas [41]	Unclear	Yes	Yes	Yes	No	No	Unclear if ITT was used, LOCF was used for missing data
Ialongo [27]	Unclear	Yes	Yes (except for greater proportion of African-American children in the placebo condition than in the high dose condition)	Yes	No	No	Not mentioned
Kelsey [42]	Unclear	Unclear	Yes	Yes	No	No	Yes and appears appropriate, LOCF was used for missing data
Kemner [22]	NA	No: open label	Yes	No	No	No	Unclear
Kollins [43]	Yes	Unclear	Yes	Yes	No	No	Yes and appears appropriate, last valid reaction time obtained post-baseline was used for missing data for primary outcome
López [44]	Unclear	Unclear	Unclear	No: single blind	No	No	Not mentioned
Martenyi [45]	Yes	Yes	Yes	Yes	No	No	Yes and appears appropriate, LOCF was used for missing data
Michelson [46]	Yes	Yes	Yes	Unclear	No	No	Efficacy measures included all randomized patients with both a baseline and a post-baseline measurement. Analyses of safety measures were restricted to randomized patients who took at least one dose of the study drug
Michelson [47]	Unclear	Unclear	Yes	Unclear	No	No	Yes and appears appropriate, LOCF was used for missing data
Montoya [48]	Yes	Yes	Yes	Yes	No	No	Yes and appears appropriate, methods used for missing data not reported
Newcorn [49]	Yes	Unclear	Yes	Yes	No	No	Yes and appears appropriate, LOCF was used for missing data
Newcorn [50]	Unclear	Unclear	Yes	Yes	No	No	Yes and appears appropriate, LOCF was used for missing data
Palumbo [51]	Yes [stratification by center (investigator) and sexual maturity status (prepubertal: Tanner stages I–II; pubertal: Tanner stages III–V)]	Yes	Yes (except for a higher percentage of whites in the clonidine group)	Yes	Yes	No	Yes and appears appropriate, LOCF was used for missing data
Pliszka [24]	Unclear	Unclear	Yes	Yes	No	No	Yes and appears appropriate, methods used for missing data not reported
Sallee [30]	Yes	Unclear	Yes	Yes	No	No	Yes and appears appropriate, LOCF was used for missing data
Shang [52]	Yes	No: open label	Yes	No: open label	No	No	Yes and appears appropriate, methods used for missing data not reported
Spencer [31]	Yes	Yes	Yes	Yes	No	No	Yes and appears appropriate, LOCF was used for missing data
Spencer [31]	Yes	Yes	Yes	Yes	No	No	Yes and appears appropriate, LOCF was used for missing data
Steele [53]	NA	No: open label	Yes	No: open label	No	No	Yes and appears appropriate, LOCF was used for missing data
Su [54]	Unclear	No: open label	No: more patients in MPH group with inattentive ADHD subtype; weight is notably higher in MPH group	No: open label	Yes: noticeably more dropouts in ATX group than in MPH group	No	Yes and appears appropriate, LOCF was used for missing data
Takahashi [55]	Yes	Yes	Yes	Yes	No	No	Yes and appears appropriate, LOCF and mean score imputation methods were used for missing data
Wang [56]	Yes	Unclear	Yes	Yes	No	No	Yes and appears appropriate, LOCF was used for missing data
Wehmeier [57]	Yes	Yes	Yes	Yes	No	No	Yes, per-protocol analysis was also done, methods used for missing data not reported
Weiss [58]	Yes	Yes	Yes	Yes	No	No	Yes and appears appropriate, LOCF was used for missing data
Wigal [59]	Unclear	Unclear	Yes	Unclear	No	No	Yes and appears appropriate, methods used for missing data not reported
Wilens [21]	Yes	Yes	Yes	Yes	Yes: notably more dropouts for PBO (around 2 × as many as for MPH)	No	Yes and appears appropriate, LOCF was used for missing data
Wilens [60]	Yes	Yes	Yes	Yes	No	No	Yes and appears appropriate, LOCF was used for missing data
Wilens [61]	Yes	Yes	Yes	Yes	No	No	Yes and appears appropriate, LOCF was used for missing data
Wolraich [62]	Yes	Unclear	Yes	Unclear	Yes	No	Unclear, LOCF was used for missing data

*ATX* atomoxetine, *ITT* intention-to-treat, *LDX* lisdexamfetamine dimesylate, *LOCF* last observation carried forward, *MPH* methylphenidate, *MPH-OROS* methylphenidate osmotic-release oral system, *NA* not available, *PBO* placebo

### Trial selection and meta-regression adjustments

Trials meeting the selection criteria were examined to determine which specific core and sensitivity analyses were feasible (Table 4). Baseline age and percent female were consistently measured for all but one study (Ialongo et al. [27]) and were available for adjustment via meta-regression in the included trials for all outcomes as well as in meta-regression adjustments in the core analyses. As Ialongo et al. [27] was missing baseline age and percent female data, it was not included in the core analyses. Baseline ADHD-RS-IV score was not reported for one GXR trial in the ADHD-RS-IV network and was not reported in a substantial number of trials in the CGI-I network. Hence, baseline ADHD-RS-IV score was only adjusted for in the sensitivity analyses and not in the core analyses. Other trial-level patient characteristics (e.g., prior treatment, comorbidities) could not be considered due to lack of data and inconsistency in definitions across trials. In the CGI-I analysis, a substantial number of studies (six) had a duration of ≤3 weeks; hence, a sensitivity analysis was conducted including these short-term studies.

**Table 4:** Sensitivity analyses by outcome

Analysis	ADHD-RS-IV change	CGI-I response	All-cause discontinuation	Discontinuation due to adverse events
Sensitivity 1	Fixed-effects model, with fixed effects for the effect of each treatment compared with placebo, and adjusted for age and percent female	Fixed-effects model, with fixed effects for the effect of each treatment compared with placebo, and adjusted for age and percent female	Fixed-effects model, with fixed effects for the effect of each treatment	Fixed-effects model, with fixed effects for the effect of each treatment
Sensitivity 2	Random-effects model, not adjusted for baseline characteristics	Random-effects model, not adjusted for baseline characteristics	Random-effects model, not adjusted for baseline characteristics	Random-effects model, not adjusted for baseline characteristics
Sensitivity 3	Fixed-effects model, not adjusted for baseline characteristics	Fixed-effects model, not adjusted for baseline characteristics	Fixed-effects model, not adjusted for baseline characteristics	Fixed-effects model, not adjusted for baseline characteristics
Sensitivity 4	Random-effects model, adjusted for age, percent female, and baseline ADHD-RS-IV score	Random-effects model, including short-term studies and adjusted for age and percent female		
Sensitivity 5	Fixed-effects model, adjusted for age, percent female, and baseline ADHD-RS-IV score	Fixed-effects model, including short-term studies and adjusted for age and percent female		

*ADHD-RS-IV* Attention-Deficit/Hyperactivity Disorder Rating Scale Version IV, *CGI-I* Clinical Global Impression–Improvement

### Analysis of efficacy

#### ADHD-RS-IV

LDX demonstrated significantly better efficacy than other treatments in terms of baseline-to-endpoint ADHD-RS-IV symptom total score change in children and adolescents. Specifically, the mean ADHD-RS-IV total score decrease from baseline relative to placebo was 14.98 (95% CrI 12.80, 17.14) for LDX, 9.33 (95% CrI 7.04, 11.63) for MPH-ER, 8.68 (95% CrI 6.72, 10.63) for GXR, and 6.88 (95% CrI 5.49, 8.22) for ATX (Table 5). There was separation in the 95% posterior CrIs of LDX compared with other treatments, and LDX had a 99.96% probability of being the most efficacious of all pharmacotherapies within the network. Among the non-stimulants, GXR was more effective than ATX with respect to ADHD-RS-IV change. Although the CrIs for GXR and ATX overlapped, the probability of GXR being more effective than ATX was 93.91%. Posterior density plots showed that the distribution of LDX was concentrated at a greater reduction in ADHD-RS-IV symptom score relative to the other treatments within the network, and that the profile of GXR visually resembled that of MPH-ER. MPH-IR was not included in this network because there were no available trials.

**Table 5:** Effect of treatment on change from baseline in ADHD-RS-IV total score (drug—placebo)

Drug	Mean change	95% credible interval	Probability of the treatment being most effective among all	Probability of GXR being more effective compared with each treatment
GXR	−8.68	(−10.63, −6.72)	<1%	–
LDX	−14.98	(−17.14, −12.80)	99.96%	<1%
ATX	−6.88	(−8.22, −5.49)	<1%	93.91%
MPH-ER	−9.33	(−11.63, −7.04)	<1%	33.04%

Core Bayesian analysis: random-effects model, combined doses, excluding short-term studies, and adjusted for age and percent female

Both forced- and optimal-dose studies were included in the network. Among forced-dose studies, randomization arms included GXR 1, 2, 3, and 4 mg/day; LDX 30, 50, and 70 mg/day; and ATX 0.5, 1.2, and 1.8 mg/kg/day. All MPH-ER studies were optimal-dose studies. MPH-IR was not included in the network because there were no available trials

*ADHD-RS-IV* Attention-Deficit/Hyperactivity Disorder Rating Scale Version IV, *ATX* atomoxetine, *GXR* guanfacine extended release, *LDX* lisdexamfetamine dimesylate, *MPH-ER* methylphenidate extended release, *MPH-IR* methylphenidate immediate release

In every sensitivity analysis, LDX continued to have the highest efficacy, with the probabilities of it being the most efficacious treatment ranging from 99.00 to 100.00%. There was no overlap with other drugs in the CrIs in any of the sensitivities, except for the random-effects model not adjusted for baseline covariates, in which there was overlap in CrIs between LDX and MPH-ER. In every sensitivity analysis, GXR was more efficacious than ATX, but with overlapping CrIs. The probability of GXR having greater efficacy than ATX remained high, ranging from 81.19 to 97.86%. In the sensitivity analysis modifying the MPH classification in Wang 2007 [56] from ER to IR, the results remained similar to those in the core analyses: the ADHD-RS-IV total score decrease from baseline relative to placebo was 10.28 (95% CrI 7.67, 12.88) for MPH-ER and 7.21 (95% CrI 3.30, 11.14) for MPH-IR, and was nearly unchanged for LDX, GXR, and ATX.

#### CGI-I

The best clinical response measured by CGI-I among all pharmacotherapies within the network was again observed in LDX: point estimates of relative risk (RR) were 2.56 (95% CrI 2.21, 2.91) for LDX, followed by 2.13 (95% CrI 1.70, 2.54) for MPH-ER, 1.94 (95% CrI 1.59, 2.29) for GXR, 1.77 (95% CrI 1.31, 2.26) for ATX, and 1.62 (95% CrI 1.05, 2.17) for MPH-IR (Table 6). The placebo risk was 0.31 (95% confidence interval [CI] 0.28, 0.34). The probability of LDX being the most efficacious drug was 96.21%. Among the non-stimulants, although the CrIs for GXR and ATX overlapped, the probability that GXR was more effective than ATX was 76.13%. Posterior density plots showed that the distribution of LDX was concentrated at a greater RR of CGI-I response than the other treatments, with relatively little overlap with other distributions.

**Table 6:** Odds ratios and relative risks for treatment response, as defined by a CGI-I score of 1 or 2 (drug versus placebo)

Drug	Odds ratio	95% credible interval for odds ratio	Probability of the treatment being most effective among all (%)	Probability of GXR being more effective compared with each treatment (%)
GXR	3.34	(2.16, 5.22)	<1	–
LDX	8.43	(4.91, 15.04)	96.21	<1
ATX	2.69	(1.52, 5.03)	<1	76.13
MPH-ER	4.27	(2.47, 7.49)	3.21	25.27
MPH-IR	2.22	(1.08, 4.45)	<1	81.68

Drug	Relative risk of drug versus placebo	95% credible interval for relative risk	Response rate	Placebo risk^a^ (95% confidence interval)
GXR	1.94	(1.59, 2.29)	0.59	0.31 (0.28, 0.34)
LDX	2.56	(2.21, 2.91)	0.79	
ATX	1.77	(1.31, 2.26)	0.54	
MPH-ER	2.13	(1.70, 2.54)	0.65	
MPH-IR	1.62	(1.05, 2.17)	0.50	

Core Bayesian analysis: random-effects model, combined doses, excluding short-term studies, and adjusted for age and percent female

Both forced- and optimal-dose studies were included in the network. Among forced-dose studies, randomization arms included GXR 1, 2, 3, and 4 mg/day; LDX 30, 50, and 70 mg/day; ATX 0.5 and 1.2 mg/kg/day; MPH-ER 20, 40, and 60 mg/day; and MPH-IR 30 mg/day. Unlike for the ADHD-RS-IV outcome, MPH-IR trial data with CGI-I scores were available. Hence, MPH-IR was included in the CGI-I network

*ADHD-RS-IV* Attention-Deficit/Hyperactivity Disorder Rating Scale Version IV, *ATX* atomoxetine, *CGI-I* Clinical Global Impression–Improvement, *GXR* guanfacine extended release, *LDX* lisdexamfetamine dimesylate, *MPH-ER* methylphenidate extended release, *MPH-IR* methylphenidate immediate release

^a^The placebo risk is the pooled risk of response (CGI-I of 1 or 2) of the placebo arms in the data. The placebo risk uncertainty is measured as the 95% confidence interval around this pooled placebo risk. The odds ratio was converted to a relative risk scale using the pooled placebo rate

In every sensitivity analysis for CGI-I response, LDX was the most efficacious treatment with probabilities ranging from 74.51 to 99.97%, although the CrIs for LDX overlapped with those of at least one other drug in all of the sensitivity analyses except for the fixed-effects model including short-term studies. MPH-ER was the second most efficacious drug in every sensitivity analysis, while GXR, ATX, and MPH-IR varied in their rankings. Among non-stimulants, GXR was observed to be more effective than ATX in every sensitivity analysis, but with overlapping CrIs. When adding studies with short-term treatment duration, the percent response of GXR over ATX increased, in terms of the probability of GXR being more effective relative to ATX. In all of the models, GXR had a high probability of being more effective than ATX, ranging from 57.16 to 98.35% in sensitivity analyses.

#### Assessment of heterogeneity

Heterogeneity was assessed in the core efficacy analyses. In the ADHD-RS-IV network, the DIC for the fixed-effects model (229.53) was lower than for the random-effects model (239.21). In the CGI-I network, the DIC for the fixed-effects model (251.65) was also lower than for the random-effects model (256.74). Thus, based on the DIC statistic [20, 64], the fixed-effects model fits the data as well or better than the random-effects model, indicating low residual heterogeneity in the meta-regression adjusted analyses. In the ADHD-RS-IV network, pairwise assessments of outcome heterogeneity based on the *I* ^2^ and *Q*-statistics did not detect statistically significant heterogeneity in 4 of the 5 pairs for which heterogeneity assessment was possible (Table 7). Pairwise heterogeneity assessments indicate the presence of heterogeneity prior to meta-regression adjustments. An *I* ^2^ of 0% indicates that there is no statistical evidence of heterogeneity; an *I* ^2^ of 70% or above suggests considerable heterogeneity may be present [64]. A significant *Q*-statistic, or one which is large in relation to degrees of freedom, can imply heterogeneity [65]. In the ADHD-RS-IV network, an *I* ^2^ of 94% (95% CI 86, 98) and *Q*-statistic of 34.39 (*p* < 0.01) was estimated in the LDX versus placebo trials. In the CGI-I network, heterogeneity assessment was possible in 6 pairwise comparisons (Table 7), of which 2 demonstrated statistically significant heterogeneity as measured by the *Q*-statistic: *I* ^2^ of 94% (95% CI not calculated) and *Q*-statistic of 16.86 (*p* < 0.01) in the LDX versus placebo trials; *I* ^2^ of 86% (95% CI not calculated) and *Q*-statistic of 7.07 (*p* < 0.01) in the ATX versus placebo trials. The results suggest that there may be outcome heterogeneity in the LDX versus placebo trials for both outcomes, and in the ATX versus placebo trials for the CGI-I score. However, the DIC assessment indicates that there is no evidence of residual heterogeneity after accounting for the meta-regression adjustments used in the analyses.

**Table 7:** Pairwise heterogeneity assessment for core analyses

Outcome	Treatments compared	Number of trials	*I* ^2^	*I* ^2^ confidence interval	*Q*-statistic	*Q* *p* value
ADHD-RS-IV	PBO, GXR	5	0.00	(0.00, 0.51)	1.68	0.79
	PBO, LDX	3	0.94	(0.86, 0.98)	34.39	<0.01
	PBO, ATX	11	0.17	(0.00, 0.57)	12.01	0.28
	PBO, MPH-ER	2	0.57	–	2.32	0.13
	ATX, MPH-ER	2	0.32	–	1.48	0.22
CGI-I	PBO, GXR	5	0.36	(0.00, 0.76)	6.22	0.18
	PBO, LDX	2	0.94	–	16.86	<0.01
	PBO, ATX	2	0.86	–	7.07	0.01
	PBO, MPH-ER	3	0.39	(0.00, 0.81)	3.27	0.20
	PBO, MPH-IR	2	0.00	–	0.27	0.60
	MPH-ER, MPH-IR	3	0.41	(0.00, 0.82)	3.38	0.18

*ADHD-RS-IV* Attention-Deficit/Hyperactivity Disorder Rating Scale Version IV, *ATX* atomoxetine, *GXR* guanfacine extended release, *LDX* lisdexamfetamine dimesylate, *MPH-ER* methylphenidate extended release, *MPH-IR* methylphenidate immediate release, *PBO* placebo

### Analysis of safety

#### All-cause discontinuation

RR estimates for all-cause discontinuation were 0.44 (95% CrI 0.25, 0.69) for MPH-IR, 0.52 (95% CrI 0.38, 0.69) for MPH-ER, 0.66 (95% CrI 0.46, 0.91) for LDX, 0.87 (95% CrI 0.66, 1.12) for GXR, and 0.88 (95% CrI 0.71, 1.08) for ATX. The placebo risk was 0.28 (95% CI 0.26, 0.30) (Table 8). The probability of MPH-IR being least likely to be discontinued was 77.23%. Among non-stimulants, the probability of GXR being more likely to be discontinued than ATX was 49.02%. The 95% CrIs and posterior density plots for these point estimates showed considerable overlap between treatments.

**Table 8:** Odds ratios and relative risks for all-cause discontinuation (drug versus placebo)

Drug	Odds ratio	95% credible interval for odds ratio	Probability of the treatment being least likely to be discontinued among all	Probability of GXR being less likely to be discontinued compared with each treatment
GXR	0.82	(0.58, 1.18)	<1%	–
LDX	0.58	(0.38, 0.88)	3.41%	8.75%
ATX	0.83	(0.63, 1.11)	<1%	50.98%
MPH-ER	0.43	(0.31, 0.62)	19.25%	<1%
MPH-IR	0.35	(0.19, 0.61)	77.23%	<1%

Drug	Relative risk of drug versus placebo	95% credible interval for relative risk	Response rate	Placebo risk^a^ (95% confidence interval)
GXR	0.87	(0.66, 1.12)	0.25	0.28 (0.26, 0.30)
LDX	0.66	(0.46, 0.91)	0.19	
ATX	0.88	(0.71, 1.08)	0.25	
MPH-ER	0.52	(0.38, 0.69)	0.15	
MPH-IR	0.44	(0.25, 0.69)	0.12	

Core Bayesian analysis: random-effects model, combined doses, excluding short-term studies, and adjusted for age and percent female

Both forced- and optimal-dose studies were included in the network. Among forced-dose studies, randomization arms included GXR 1, 2, 3, and 4 mg/day; LDX 30, 50, and 70 mg/day; ATX 0.5, 1.2, and 1.8 mg/kg/day; MPH-IR 0.4 and 0.8 mg/kg/day; and MPH-ER 18, 20, and 36 mg/day. Unlike for the ADHD-RS-IV outcome, MPH-IR trial data with all-cause discontinuation rates were available. Hence, MPH-IR was included in the all-cause discontinuation network

*ADHD-RS-IV* Attention-Deficit/Hyperactivity Disorder Rating Scale Version IV, *ATX* atomoxetine, *GXR* guanfacine extended release, *LDX* lisdexamfetamine dimesylate, *MPH-ER* methylphenidate extended release, *MPH-IR* methylphenidate immediate release

^a^The placebo risk is the pooled risk of discontinuation of the placebo arms in the data. The placebo risk uncertainty is measured as the 95% confidence interval around this pooled placebo risk. The odds ratio was converted to a relative risk scale using the pooled placebo rate

In all sensitivity analyses, MPH-IR continued to be the treatment least likely to be discontinued due to any cause, with probabilities ranging from 84.16 to 92.40%. MPH-IR was followed in all-cause discontinuation by MPH-ER and LDX, although CrIs overlapped between all drugs. While the core analysis found ATX to be most likely drug to be discontinued, followed by GXR, the sensitivity analyses found GXR to be the most likely to be discontinued, followed by ATX. The probability of GXR being more likely to be discontinued than ATX ranged from 54.03 to 94.77% in sensitivity analyses. In the sensitivity analysis modifying the MPH classification in Wang 2007 [56] from ER to IR, all findings remained nearly unchanged relative to those in the core analyses.

#### Discontinuation due to AEs

RR estimates for AE-related discontinuation were 1.20 (95% CrI 0.32, 3.06) for MPH-IR, 1.38 (95% CrI 0.60, 2.68) for MPH-ER, 2.39 (95% CrI 1.26, 4.11) for ATX, 3.11 (95% CrI 1.20, 6.76) for LDX, and 4.49 (95% CrI 2.10, 8.81) for GXR. The placebo rate was 0.02 (95% CI 0.01, 0.02) (Table 9). The probability of MPH-IR being the least likely drug to be discontinued was 63.85%. Among non-stimulants, the probability of GXR being more likely to be discontinued than ATX was 92.26%. The 95% CrIs for all treatments overlapped and density plots of the posterior samples also showed considerable overlap in the distributions for all the treatments. The number of discontinuation events for MPH-IR was small (9 events); this led to skewed posterior distributions and high levels of statistical uncertainty for this drug.

**Table 9:** Odds ratios and relative risks for discontinuation due to AEs (drug versus placebo)

Drug	Odds ratio	95% credible interval for odds ratio	Probability of the treatment being least likely to be discontinued among all	Probability of GXR being less likely to be discontinued compared with each treatment
GXR	4.50	(2.14, 10.31)	<1%	–
LDX	2.95	(1.21, 7.59)	1.67%	22.55%
ATX	2.35	(1.27, 4.37)	<1%	7.74%
MPH-ER	1.29	(0.59, 2.77)	33.50%	1.05%
MPH-IR	1.02	(0.32, 3.18)	63.85%	1.89%

Drug	Relative risk of drug versus placebo	95% credible interval for relative risk	Response rate	Placebo risk^a^ (95% confidence interval)
GXR	4.49	(2.10, 8.81)	0.08	0.02 (0.01, 0.02)
LDX	3.11	(1.20, 6.76)	0.06	
ATX	2.39	(1.26, 4.11)	0.04	
MPH-ER	1.38	(0.60, 2.68)	0.03	
MPH-IR	1.20	(0.32, 3.06)	0.02	

Core Bayesian analysis: random-effects model, combined doses, excluding short-term studies, and adjusted for age and percent female

Both forced- and optimal-dose studies were included in the network. Among forced-dose studies, randomization arms included GXR 1, 2, 3, and 4 mg/day; LDX 30, 50, and 70 mg/day; ATX 0.5, 1.2, and 1.8 mg/kg/day; and MPH-ER 18, 20, and 36 mg/day. Unlike for the ADHD-RS-IV outcome, MPH-IR trial data with AE-related discontinuation rates were available. Hence, MPH-IR was included in the AE-related discontinuation network. All MPH-IR studies were optimal-dose studies

*ADHD-RS-IV* Attention-Deficit/Hyperactivity Disorder Rating Scale Version IV, *AE* adverse event, *ATX* atomoxetine, *GXR* guanfacine extended release, *LDX* lisdexamfetamine dimesylate, *MPH-ER* methylphenidate extended release, *MPH-IR* methylphenidate immediate release

^a^The placebo risk is the pooled risk of discontinuation of the placebo arms in the data. The placebo risk uncertainty is measured as the 95% confidence interval around this pooled placebo risk. The odds ratio was converted to a relative risk scale using the pooled placebo rate

In all sensitivity analyses, MPH-IR continued to be the treatment least likely to be discontinued in terms of AE-related discontinuation, with probabilities ranging from 62.90 to 73.18%. MPH-IR was followed in tolerability by MPH-ER, ATX, LDX, and GXR in all sensitivity analyses, except for the fixed-effects adjusted model, where LDX had a lower RR than ATX; CrIs overlapped between all drugs in the sensitivity analyses. The probability of GXR being more likely to be discontinued than ATX ranged from 90.39 to 96.87% in sensitivity analyses. In the sensitivity analysis modifying the MPH classification in Wang 2007 [56] from ER to IR, all findings remained nearly unchanged relative to those in the core analyses.

## Discussion

To our knowledge, this is the first systematic comparison of the safety and efficacy of GXR with other pharmacotherapies available, indicated, or frequently used to treat children and adolescents with ADHD within a network meta-analysis and MTC. This comparison of pharmacotherapies for children and adolescents with ADHD found that LDX was more effective than other treatments, including MPH-ER, with a clear separation in its 95% posterior distribution CrIs when comparing baseline-to-endpoint ADHD-RS-IV total score change. In the CGI-I comparison, LDX had a higher rate of response than other treatments, but the posterior 95% CrIs for the RR overlapped with those for MPH-ER. Among the non-stimulants included, GXR was more effective than ATX when comparing ADHD-RS-IV total score change and CGI-I response, although the 95% CrIs for efficacy measures overlapped. GXR was also shown to have a higher probability of being more efficacious than ATX, although the 95% CrIs and posterior distributions again overlapped. MPH-ER demonstrated higher efficacy than GXR on the ADHD-RS-IV and CGI-I measures, but 95% CrIs and posterior distributions showed overlap between the two treatments. This indicates that the level of evidence on the efficacy of the studied treatments for these outcomes may approach level 1a [66]. In the core safety assessments, GXR and ATX appeared similar in the evaluation of all-cause discontinuation, but ATX had a lower RR of AE-related discontinuation, although the 95% CrIs and posterior distributions overlapped. While this study focused on efficacy and safety, a risk–benefit assessment would be a useful consideration in deciding how these results may be applied in real-world practice, and this could be a useful topic for further research.

Although we found that GXR had higher rates of CGI-I response than MPH-IR, all three MPH-IR studies included in the CGI-I efficacy MTC were published during or before 2006 [26, 59, 62]. The timing of these studies might have influenced their design or conduct, and hence modified the relative rates of CGI-I response for MPH-IR versus placebo, explaining the higher CGI-I response rates of GXR in comparison with MPH-IR. The timing of the studies might also have influenced their choice of outcome (all three studies used the Inattention/Overactivity With Aggression Conners’ Rating Scale or SNAP-IV, rather than ADHD-RS-IV), leading to exclusion of MPH-IR as a comparator in the ADHD-RS-IV network for efficacy analysis due to a lack of data.

Heterogeneity in the evidence network for the efficacy analyses was assessed using methods recommended by NICE guidelines [20]. The DIC of the fixed and random-effects models were compared; this assessment found that there is no statistical evidence of residual heterogeneity after the meta-regression adjustments. In addition, for completeness, following the NICE guidelines, heterogeneity was also assessed using *I* ^2^ and Cochran’s *Q* test for each feasible pair of medications without regard to the meta-regression adjustments. These two tests showed no statistically significant evidence of heterogeneity for ADHD-RS-IV change from baseline and CGI-I for pairwise comparisons of GXR versus placebo, ATX versus placebo, MPH-ER versus placebo, MPH-ER versus ATX, MPH-IR versus placebo, and MPH-ER versus MPH-IR, indicating that variability in effect-size estimates for these comparisons was due to sampling error within studies, rather than heterogeneity. Statistically significant heterogeneity was detected for LDX versus placebo for ADHD-RS-IV change from baseline and for LDX versus placebo and ATX versus placebo for CGI-I. This indicates that the treatment effect may differ in magnitude across trials for the LDX versus placebo and ATX versus placebo pairwise comparisons without the meta-regression adjustments. Overall, the heterogeneity assessment found no heterogeneity in the evidence network for the efficacy analyses when accounting for meta-regression adjustments.

The findings from the core analyses that LDX was the most efficacious treatment and MPH-IR the most tolerable were confirmed by all of the sensitivity analyses across efficacy and safety outcomes. Among non-stimulants, GXR was more efficacious than ATX in all core and sensitivity analyses for the ADHD-RS-IV and CGI-I outcomes. Core and sensitivity analyses for AE-related discontinuation were consistent in showing that ATX had higher tolerability than GXR. While the core analysis for all-cause discontinuation found ATX to have the highest RR of discontinuation, followed by GXR, the three sensitivity analyses all found GXR to have the highest RR, followed by ATX. There was a large overlap in the CrIs for both safety outcomes. These differences are potentially related to differences in the two drugs’ mechanism of action: GXR is a selective α-2 adrenergic agonist, while ATX is a norepinephrine reuptake inhibitor.

A systematic literature review and meta-analysis of studies comparing GXR and placebo was conducted recently by Ruggiero et al. [7], but the current study has important differences. Ruggiero et al. performed direct comparisons solely on GXR (by pooling GXR and placebo arms) and did not supplement the information using clinical study reports; in contrast, the current study included a broader selection of pharmacotherapies and considered both direct and indirect evidence, and used information from clinical study reports to supplement the primary publication, which allowed inclusion of the ADHD-RS-IV change from baseline from Biederman et al. [28] and Sallee et al. [30]. In these cases, the clinical study reports were used to prevent any potential bias resulting from exclusion of this information. We excluded the following studies that were included in the publication by Ruggiero et al. [7]: Scahill et al. [67] only included subjects from a specialty tic disorder clinic; Connor et al. [68] required all subjects to have oppositional symptoms; and trial NCT01081132 did not have any publications. This allowed our study to be more homogenous and to focus solely on ADHD, whereas Ruggiero et al. [7] included two common comorbidities. The present study assessed somewhat different outcomes than Ruggiero et al. [7], which assessed CGI-I response, rates of patients with at least one AE, and rates of individual AEs. Nevertheless, our conclusions were generally consistent with those of Ruggiero et al. [7] in the common analysis of CGI-I response. In their CGI-I analysis, Ruggiero et al. [7] found that GXR was associated with a greater proportion of patients with CGI-I response, with a pooled odds ratio (OR) of 3.18 (95% CI 2.44, 4.13), with between-study heterogeneity accounting for 26% of the difference. The core analysis of CGI-I in the present study found a comparable OR of 3.34 (95% CrI 2.16, 5.22) for GXR.

A recent systematic review and meta-analysis, by Stuhec and colleagues [69], included LDX, MPH, and bupropion, but not GXR. The study incorporated data from 28 trials using the random-effects model of derSimonian and Laird [70] and compared each drug with placebo; it did not incorporate MTCs. ORs for all-cause discontinuation reported by Stuhec et al. [69] are comparable with those in this study for ATX (OR: 0.91 [95% CI 0.66, 1.24] in Stuhec et al. [68] versus 0.83 [95% CrI 0.63, 1.11]) and LDX (OR: 0.6 [95% CI 0.22, 1.65] versus 0.58 [95% CrI 0.38, 0.88]). Other outcomes and treatments were reported in ways directly comparable with this study. Roskell et al. [71] is another recent systematic literature review and indirect comparison. Unlike the preceding two comparisons and the present one, Roskell et al. [71] focused on comparing LDX with MPH and ATX. The study identified 32 trials. While the results were not directly comparable with those of the present study, which compares the treatments with placebo rather than LDX, the approximate RR for CGI-I response was 0.65 for ATX versus LDX in Roskell et al. [71], compared with 0.69 in the present study (obtained as the ratio of the RRs for ATX versus placebo and LDX versus placebo). The corresponding RR comparison (Roskell et al. [71] versus present study) is 1.33 versus 1.33 for all-cause discontinuation and 0.58 versus 0.77 for AE-related discontinuation. Thus, the findings of the Roskell et al. study are consistent with those of the present study.

Furthermore, pooled results of direct and indirect evidence with respect to the non-stimulants GXR and ATX from this MTC were also consistent with previously published direct comparisons. Direct evidence was available from Hervas et al. [41], in which a comparison between GXR and the ATX reference arm was pre-specified; this comparison was carried out for health technology assessment purposes. That article reported ADHD-RS-IV total score changes from baseline (standard deviation) in the placebo, GXR, and ATX arms of −15.0 (13.07), −23.9 (12.41), and −18.6 (11.91), respectively. There was a significant difference between GXR and ATX when analysing the difference in mean change from baseline in ADHD-RS-IV total score at the trial endpoint (treatment effect: −5.1 [95% CI −8.2, −2.0], *p* = 0.001) favoring GXR (secondary analyses, not controlled for multiplicity). The results from that trial are broadly consistent with this MTC, in that GXR exhibited a larger treatment effect than ATX. In addition, Hervas et al. [41] reported the proportion of patients achieving a CGI-I response in each of three arms: placebo, GXR, and ATX. The percentages in these arms were 44.1, 67.9, and 56.3%, respectively. The raw RR of CGI-I compared with placebo, based on these proportions, was 1.54 for GXR and 1.28 for ATX. The ratio of RRs (RR for GXR/RR for ATX) was larger in the Hervas et al. study [41] than in this MTC (1.21 versus 1.09). As in this MTC, GXR exhibited a numerically greater treatment effect than ATX in the study by Hervas et al. [41]. Lastly, these results are also supportive of published indirect comparisons of ATX versus GXR [5, 72]. In the study by Signorovitch et al. [72], GXR was found to be associated with a significantly greater mean reduction in the CPRS-short form oppositional subscale score compared with ATX when comparing matched populations (GXR: −5.0 [95% CI −6.6, −3.4]; ATX: −2.4 [95% CI −3.7, −1.1], *p* = 0.01). In the study by Sikirica et al. [5], GXR was found to be associated with a significantly greater mean reduction in ADHD-RS-IV total score compared with ATX when comparing matched populations (−7.0 [standard error: 2.2], *p* < 0.01). Significantly greater reductions with GXR over ATX were also found in scores on the ADHD-RS-IV hyperactivity/impulsivity (−3.8 [standard error: 1.2], *p* < 0.01) and inattention (−3.2 [standard error: 1.3], *p* < 0.05) subscales.

Regarding safety results, we found that all of the treatments were generally similar in their all-cause and AE-related discontinuation rates, with no clear differences between treatments. There seemed to be a slightly larger probability of more discontinuations in the GXR group due to AEs relative to other treatments. However, further study is needed to confirm if this is a real finding or one related to the fact that safety is often measured as a proportion rather than a continuous measure, which further limits the analysis and interpretability as this effectively gives a smaller number of observations from these RCTs for safety than for efficacy analyses.

There were several limitations to this study due to data constraints. The analyses adjusted for baseline age, sex, and disease severity but, as is common to meta-analyses, differences that were not uniformly reported across the trials (e.g., prior treatments, comorbidities) and heterogeneity due to unobserved variables could not be captured. This may have led to uncontrolled residual confounding. Matching-adjusted indirect comparisons [5, 72] can increase the validity of comparison when individual patient-level data are available for some of the trials. Heterogeneity could not be assessed in this MTC by comparing placebo effects because the methodology, as is common in MTCs, does not compare different placebo arms. However, an assessment comparing the DIC between the fixed- and random-effects analyses indicated that heterogeneity was not statistically significant in the evidence networks for the efficacy outcomes. This analysis included trials of 3–16 weeks duration. These cutoffs were chosen to allow sufficient time for the effects of different treatments to take effect while still retaining comparability of trials. The analysis did not adjust for differences in trial length, under the assumption that trial durations were chosen by investigators according to each drug’s duration of action. However, variation in trial durations might have had unknown effects on the outcomes of this analysis. For example, adverse effects may take longer to occur with some drugs than with others [73]. In such cases, the drugs whose effects manifest earlier will have an unfair disadvantage in this type of study, due to the large number of studies of relatively short duration. However, the findings in sensitivity analyses including shorter duration studies were consistent with those in the core analyses. Separate comparisons for stimulant-naïve and stimulant-failed populations were not feasible due to data limitations. Further research is needed to identify subgroups of patients who might receive greater benefit from specific treatments once more indirect or preferably direct head-to-head evidence is available. For example, prior treatment might indicate refractory patients in whom a reduced treatment effect is likely. Furthermore, analyses of discontinuation rates might not be directly interpretable as indications of drug safety, as discontinuation can be much more directly influenced by trial protocol specifications than efficacy measurements. Different trials might not be consistent in their discontinuation specifications—in particular, AE-related discontinuation rates did not account for different types of AEs. AE-related discontinuation also had limited sample sizes for MPH-IR. This study relied on assessments of each trial to account for patients who dropped out for any reason (including discontinuation of study medication); for example, trials in which patients dropped out were not excluded. This study used the Bayesian method, as suggested in the NICE Decision Support Unit Technical Support Documents; however, the results from this method might depend on the choice of prior distributions for the models’ parameters. Non-informative priors were chosen so that the priors would not influence the results, and sensitivity analyses indicated that the results were stable with respect to priors. This study also focused on each of the included treatments as monotherapy, and its findings are not generalizable to combination-therapy settings. In addition, this study treated all versions of a particular drug, for example MPH-ER, as the same, while in reality they might have different effects. It also treated all doses of any specific drug as the same; thus, the efficacy and safety findings apply to a population in which proportions of patients receive different doses of each drug. The study also did not distinguish between studies that reported doses using different units or distinguish between those with and without a titration period, due to the sparsity of data within these categories. The inclusion of open-label trials may have led to biases favoring ATX [69]. Another difference that was not specifically assessed within the scope of the study was whether the trials were laboratory trials. For 4 RCTs, the MPH duration of action was not clearly specified; studies were classified as MPH-ER or -IR based on dosing details provided in the corresponding publications. Because the classification into ER or IR is not provided in the publications, there is potential for inaccuracies in the classification for these 4 studies. A misclassification in these studies is unlikely to have much impact on the findings of the meta-analysis, however, because the classification was performed for only 4 out of 20 MPH studies, and the studies had small to moderate sample sizes. In a sensitivity analysis changing the imputed classification of MPH in the largest of these 4 studies, there was little change in the results, showing that the findings are robust to this imputation. Finally, lack of data limited the treatments that could be included in the MTC, as well as the outcome measures that could be used for the comparisons. In particular, d-AMPH, CIR, and GIR could not be included even though these drugs have similarities to GXR, as they did not form a connected network with GXR through any of the publications identified.

In summary, based on published evidence, both direct and indirect, this study found that LDX had greater efficacy than GXR, ATX, and MPH in reducing symptoms in children and adolescents with ADHD, with no overlap in CrIs on the ADHD-RS-IV. The probability was also higher in the overall impression of improvement, although there was overlap in CrIs. Safety, as measured by all-cause and AE-related discontinuations, was slightly better for MPH relative to other therapies, but the sample sizes were relatively low and statistical uncertainty was high for this outcome. Further study with an updated network is warranted if additional direct or indirect evidence becomes available.

## Electronic supplementary material

Below is the link to the electronic supplementary material.

Supplementary material 1 (DOCX 106 kb)

Supplementary material 2 (DOCX 50 kb)
