# Supplementary material for: Comparative efficacy and safety of attention-deficit/hyperactivity disorder pharmacotherapies, including guanfacine extended release: a mixed treatment comparison
Source: Eur Child Adolesc Psychiatry. 2017 Mar 3;26(8):875–97. doi: 10.1007/s00787-017-0962-6 (PMC5532417; doi:10.1007/s00787-017-0962-6)
Supplement: Supplementary file 1 — Supplementary material 1 (DOCX 106 kb) [file 787_2017_962_MOESM1_ESM.docx]

|  |
| --- |
| Study Protocol for Systematic Literature Review of Clinical Trials in Children and Adolescents with Attention Deficit Hyperactivity Disorder |
|  |
|  |
| **9/26/2016** |

Table of Contents

[Databases 2](#_Toc462240394)

[Published research 2](#_Toc462240395)

[Conference proceedings 2](#_Toc462240397)

[Search Strategies 4](#_Toc462240398)

[1. Published Research 4](#_Toc462240399)

[Table 1A. MEDLINE, MEDLINE In-Process, PsycINFO, Embase, and Cochrane (Ovid) Search Strategy 4](#_Toc462240400)

[Table 1B. CINAHL (EBSCO) Search Strategy 15](#_Toc462240401)

[Table 1C. Science Citation Index (SCI) [Web of Knowledge] Search Strategy 26](#_Toc462240402)

[Inclusion and Exclusion Criteria 35](#_Toc462240403)

[Table 2: Inclusion and exclusion criteria of the systematic search 36](#_Toc462240404)

**Study Protocol for Systematic Literature Review of Clinical Trials in Children and Adolescents with Attention Deficit Hyperactivity Disorder^[[1]](#footnote-2)^**

# Databases

1. The specific databases that will be searched and the service providers that will be used

A series of searches will be undertaken in the following databases to identify studies of drug therapies in children and adolescents with attention deficit hyperactivity disorder (ADHD), which could be used to provide evidence on clinical effectiveness and basis for mixed treatment comparison (MTC) in children and adolescents with ADHD. The databases are proposed based on the NICE Guidance.^[[2]](#footnote-3)^

The search will include:

### Published research

- MEDLINE and MEDLINE In-Process (OvidSP*, ovidsp.ovid.com*)
- EMBASE (OvidSP)
- PsycINFO (OvidSP)
- Cochrane Central Register of Controlled Trials (CENTRAL) (Cochrane Library, OvidSP)
- CINAHL (EBSCO, *search.ebscohost.com*)
- Science Citation Index (SCI, *www.thomsonreuters.com/web-of-science*)

### Conference proceedings

- The EMBASE search covers many conferences, for example:
  - International / World Congress on ADHD, hosted by the World Federation ADHD (*www.adhd-congress.or*g)
  - European Congress of Psychiatry (*www.epa-congress.org*)
- Additional conference proceedings of interest from 2012-2016 will be captured by searching the following conference databases:
  - American Academy of Child and Adolescent Psychiatry (AACAP) Annual Meeting (*www.aacap.org*)
  - European Society of Child and Adolescent Psychiatry (ESCAP) Congress (*www.escap.eu*)

1. The date on which the search will be conducted

All searches will be conducted between May 20 and May 26, 2016. Webpages or PDF which document the conducted search will be saved for records.

1. The date span of the search

The date span of the searches is reported below for each database:

- MEDLINE (1946-May, 2016)
- MEDLINE In-Process (up to May, 2016)
- EMBASE (1974- May, 2016)
- PsycINFO (1806 - May, 2016)
- Cochrane Central Register of Controlled Trials (CENTRAL) (up to May, 2016)
- CINAHL (EBSCO) (1937 – May, 2016)
- Science Citation Index (SCI) (1993-May, 2016)

The current search strategy is designed to balance comprehensiveness and efficiency according to previously published HTA reviews and approaches used by NICE in MTC.

1. The complete search strategies proposed in Table 1A-1C will be used, including combinations of the search terms: text words (free text), subject index headings (for example, MeSH) and the relationship between the search terms (for example, Boolean).

# Search Strategies

## 1. Published Research

### Table 1A. MEDLINE, MEDLINE In-Process, PsycINFO, Embase, and Cochrane (Ovid) Search Strategy

| **Term Group** | **Search Number** | **Search Terms** | | |
| --- | --- | --- | --- | --- |
| **Population** | #1-23 | 1 | exp "Attention Deficit and Disruptive Behavior Disorders"/ or exp "Attention Deficit Disorder with Hyperactivity"/ |  |
|  |  | 2 | attention deficit hyperactivity disorder.mp |  |
|  |  | 3 | attention deficit disorder$.mp |  |
|  |  | 4 | attention-deficit hyperactivity disorder.mp |  |
|  |  | 5 | adhd.mp |  |
|  |  | 6 | ad hd.mp |  |
|  |  | 7 | addh.mp |  |
|  |  | 8 | hyperkinetic disorder.mp |  |
|  |  | 9 | (attention deficit AND disruptive behavior$).mp |  |
|  |  | 10 | hyperactiv$.ti |  |
|  |  | 11 | disruptiv$.ti |  |
|  |  | 12 | impulsiv$.ti |  |
|  |  | 13 | inattentiv$.ti |  |
|  |  | 14 | inattention$.ti |  |
|  |  | 15 | hyperkin$.ti |  |
|  |  | 16 | hyper kin$.ti |  |
|  |  | 17 | hkd.ti |  |
|  |  | 18 | minimal brain dysfunction.ti |  |
|  |  | 19 | hyperkinetic syndrome.mp |  |
|  |  | 20 | attention deficit$.mp |  |
|  |  | 21 | attention disturbance.mp |  |
|  |  | 22 | disruptive behavior.mp |  |
|  |  | 23 | OR/1-22 |  |
| **Interventions** |  |  |  |  |
| Methylphenidate | #24-59 | 24 | Methylphenidate/ |  |
|  |  | 25 | Ritalin/ |  |
|  |  | 26 | (Ritalin adj (SR OR LA)).mp |  |
|  |  | 27 | ritaline/ |  |
|  |  | 28 | equasym/ |  |
|  |  | 29 | (equasym adj XL).mp |  |
|  |  | 30 | centedrin/ |  |
|  |  | 31 | phenidylate/ |  |
|  |  | 32 | tsentedrin/ |  |
|  |  | 33 | d erythro methyl phenidylacetate.mp |  |
|  |  | 34 | alpha phenyl alpha 2 piperidly acetic acid methyl ester.mp |  |
|  |  | 35 | alpha phenyl 2 piperidineacetic acid methyl ester.mp |  |
|  |  | 36 | d erythro methyl phenidylacetate.mp |  |
|  |  | 37 | d1 erythro methyl phenidylacetate.mp |  |
|  |  | 38 | (metadate OR (medadate adj (ER OR CD))).mp |  |
|  |  | 39 | methylfenidate.mp |  |
|  |  | 40 | methyl phenidate.ti,ab |  |
|  |  | 41 | methylphenidylacetate.ti,ab |  |
|  |  | 42 | methylphenindate.ti,ab |  |
|  |  | 43 | methylphenydate.mp |  |
|  |  | 44 | methyl 2 phenyl 2 piperid 2 ylacetate.mp |  |
|  |  | 45 | phenidylate.mp |  |
|  |  | 46 | phenidyl hydrochloride.mp |  |
|  |  | 47 | (sr 20 or sr20).mp |  |
|  |  | 48 | (Methylin or (methylin adj ER)).mp. |  |
|  |  | 49 | Attenta.mp |  |
|  |  | 50 | Riphenidate.mp |  |
|  |  | 51 | Ritalina.mp |  |
|  |  | 52 | rubifen.mp |  |
|  |  | 53 | tranquilyn.mp |  |
|  |  | 54 | (medikinet OR (medikinet adj XL)).mp |  |
|  |  | 55 | concerta.mp |  |
|  |  | 56 | (Focalin OR (Focalin adj XR)).mp |  |
|  |  | 57 | daytrana.mp |  |
|  |  | 58 | (Quillivant OR (Quillivant adj ER)).mp |  |
|  |  | 59 | OR/24-58 |  |
|  |  |  |  |  |
| Atomoxetine | #60-78 | 60 | Atomoxetin$.mp |  |
|  |  | 61 | Atomoxetine/ |  |
|  |  | 62 | Tomoxetine.mp |  |
|  |  | 63 | ly 139602.mp |  |
|  |  | 64 | ly 139603.mp |  |
|  |  | 65 | ly139602.mp |  |
|  |  | 66 | ly139603.mp |  |
|  |  | 67 | n methyl gamma 2 methylphenoxy phenylpropylamine.mp |  |
|  |  | 68 | n methyl gamma 2 methylphenoxy benzenepropanamine.mp |  |
|  |  | 69 | n methyl 3 2 methylphenoxy 3 phenylpropylamine.mp |  |
|  |  | 70 | n methyl 3 phenyl 3 ortho tolyloxy propylamine.mp |  |
|  |  | 71 | Strattera.mp |  |
|  |  | 72 | Recit.mp |  |
|  |  | 73 | norepinephrine reuptake inhibitor.mp |  |
|  |  | 74 | 83015 26 3.rn,mp. |  |
|  |  | 75 | 82248 59 7.rn,mp. |  |
|  |  | 76 | 82857 39 4.rn,mp. |  |
|  |  | 77 | 82857 40 7.rn,mp. |  |
|  |  | 78 | OR/60-77 |  |
|  |  |  |  |  |
| Dexamphetamine | #79-138 | 79 | dextroamphetamine/ |  |
|  |  | 80 | dexamphetamine.mp |  |
|  |  | 81 | dexamfetamine.mp |  |
|  |  | 82 | d amphetamine.mp |  |
|  |  | 83 | (Dexedrine OR (Dexedrine adj SR) OR (Dexedrine adj Spansules)).mp |  |
|  |  | 84 | (Dextroamphetamine OR (dextroamphetamine adj ER)).mp |  |
|  |  | 85 | dextro amphetamine.mp |  |
|  |  | 86 | afatin.mp |  |
|  |  | 87 | Afettine.mp |  |
|  |  | 88 | Albemap.mp |  |
|  |  | 89 | amfetasul.mp |  |
|  |  | 90 | amitrene.mp |  |
|  |  | 91 | amphedrine.mp |  |
|  |  | 92 | amphex.mp |  |
|  |  | 93 | amsustain.mp |  |
|  |  | 94 | ardex.mp |  |
|  |  | 95 | betafedrina.mp |  |
|  |  | 96 | betaphedrine.mp |  |
|  |  | 97 | biphetamine.mp |  |
|  |  | 98 | carboxyphen.mp |  |
|  |  | 99 | dadex.mp |  |
|  |  | 100 | methylphenethylamin.mp |  |
|  |  | 101 | (d alpha adj2 methylphenethylamin$).mp |  |
|  |  | 102 | d alpha methylphenethylamine sul?ate.mp |  |
|  |  | 103 | Daprisal.mp |  |
|  |  | 104 | d beta phenylisopropylamine.mp |  |
|  |  | 105 | dephadren.mp |  |
|  |  | 106 | dexadrine.mp |  |
|  |  | 107 | Dexaline.mp |  |
|  |  | 108 | dexalme.mp |  |
|  |  | 109 | dexalone.mp |  |
|  |  | 110 | dexamed.mp |  |
|  |  | 111 | dexamphetamine.mp |  |
|  |  | 112 | dexamphethamine.mp |  |
|  |  | 113 | dexamphoid.mp |  |
|  |  | 114 | dexamyl.mp |  |
|  |  | 115 | (dexaspan adj b).mp |  |
|  |  | 116 | dexeamphetanine.mp |  |
|  |  | 117 | dexoval.mp |  |
|  |  | 118 | dextrostat.mp |  |
|  |  | 119 | diocarb.mp |  |
|  |  | 120 | diocurb.mp |  |
|  |  | 121 | Domafate.mp |  |
|  |  | 122 | Domefate.mp |  |
|  |  | 123 | Doxedrine.mp |  |
|  |  | 124 | Dexmethylphenidate.mp |  |
|  |  | 125 | d 1 phenyl 2 aminopropane.mp |  |
|  |  | 126 | dynaphenyl.mp |  |
|  |  | 127 | evrodex.mp |  |
|  |  | 128 | hetamine.mp |  |
|  |  | 129 | nsc 73713.mp |  |
|  |  | 130 | obesedrin.mp |  |
|  |  | 131 | obesonil.mp |  |
|  |  | 132 | phetadex.mp |  |
|  |  | 133 | (simpamina adj d).mp |  |
|  |  | 134 | sympamin.mp |  |
|  |  | 135 | LiquADD.mp |  |
|  |  | 136 | 51 64 9.rn,mp |  |
|  |  | 137 | Attentin.mp |  |
|  |  | 138 | OR/79-137 |  |
|  |  |  |  |  |
| Lisdexamfetamine / Lisdexamphetamine | #139-155 | 139 | Lisdexam*etamine dimes?late.mp. |  |
|  |  | 140 | Lisdexam*etamine.mp |  |
|  |  | 141 | lis-dexam*etamine dimes?late.mp |  |
|  |  | 142 | lisdexam*etamine mes?late.mp |  |
|  |  | 143 | Lisdexamfetamine/ |  |
|  |  | 144 | Lisdexamphetamine/ |  |
|  |  | 145 | Vyvanse.mp |  |
|  |  | 146 | Elvanse.mp |  |
|  |  | 147 | NRP104.mp |  |
|  |  | 148 | NRP 104.mp |  |
|  |  | 149 | SPD 489.mp |  |
|  |  | 150 | SPD489.mp |  |
|  |  | 151 | 608137 32 2.rn,mp |  |
|  |  | 152 | 608137 33 3.rn,mp |  |
|  |  | 153 | 819871 04 0.rn,mp |  |
|  |  | 154 | lysine 1 methyl phenylethylamide.mp |  |
|  |  | 155 | OR/139-154 |  |
|  |  |  |  |  |
| Guanfacine (extended release /immediate release) | #156-174 | 156 | Intuniv.mp |  |
|  |  | 157 | Intuniv/ |  |
|  |  | 158 | guanfacin$.mp |  |
|  |  | 159 | guanfascin$.mp |  |
|  |  | 160 | guarfacin$.mp |  |
|  |  | 161 | guanfacine/ |  |
|  |  | 162 | ((N adj amidino adj #2 OR #2 adj #6 adj dichlorophenyl) AND acetamide).mp |  |
|  |  | 163 | ((N adj aminoiminomethyl #2 adj #6 adj dichloro) AND benzeneacetamide).mp |  |
|  |  | 164 | Lon 798.mp |  |
|  |  | 165 | BS-100-141.mp |  |
|  |  | 166 | BS 100 141.mp |  |
|  |  | 167 | BS100141.mp |  |
|  |  | 168 | SPD 503.mp |  |
|  |  | 169 | SPD503.mp |  |
|  |  | 170 | 29110 47 2.rn,mp |  |
|  |  | 171 | Tenex.mp |  |
|  |  | 172 | Estulic.mp |  |
|  |  | 173 | Dipresan.mp |  |
|  |  | 174 | OR/156-173 |  |
|  |  |  |  |  |
| Clonidine (immediate release) | #175-198 | 175 | clonidine/ |  |
|  |  | 176 | 4205-90-7.rn,mp |  |
|  |  | 177 | alpha-2 adrenergic agonist.mp |  |
|  |  | 178 | adrenergic alpha-2 receptor agonist.mp |  |
|  |  | 179 | ST 155.mp |  |
|  |  | 180 | ST155.mp |  |
|  |  | 181 | M 5041T.mp |  |
|  |  | 182 | M5041T.mp |  |
|  |  | 183 | Catapres.mp |  |
|  |  | 184 | Catapresan.mp |  |
|  |  | 185 | Catapressan.mp |  |
|  |  | 186 | Chlophazolin.mp |  |
|  |  | 187 | Clofelin.mp |  |
|  |  | 188 | Clofenil.mp |  |
|  |  | 189 | Clopheline.mp |  |
|  |  | 190 | Dixarit.mp |  |
|  |  | 191 | Gemiton.mp |  |
|  |  | 192 | Hemiton.mp |  |
|  |  | 193 | Isoglaucon.mp |  |
|  |  | 194 | Klofelin.mp |  |
|  |  | 195 | Klofenil.mp |  |
|  |  | 196 | Jenloga.mp |  |
|  |  | 197 | (Imidazol 2 amine N 2 6 dichlorophenyl 4 5 dihydro).mp |  |
|  |  | 198 | OR/175-197 |  |
|  |  |  |  |  |
| **All interventions** |  | 199 | 59 or 78 or 138 or 155 or 174 or 198 |  |
|  |  |  |  |  |
| **All populations and interventions** |  | 200 | 23 AND 199 |  |
|  |  |  |  |  |
| **MESHED SIGN, RCT^[[3]](#footnote-4)^** | #201-243 | 201 | Randomized Controlled Trials as Topic/ |  |
|  |  | 202 | randomized controlled trial/ |  |
|  |  | 203 | Random Allocation/ |  |
|  |  | 204 | Double Blind Method/ |  |
|  |  | 205 | Single Blind Method/ |  |
|  |  | 206 | clinical trial/ |  |
|  |  | 207 | clinical trial, phase i.pt. |  |
|  |  | 208 | clinical trial, phase ii.pt. |  |
|  |  | 209 | clinical trial, phase iii.pt. |  |
|  |  | 210 | clinical trial, phase iv.pt. |  |
|  |  | 211 | controlled clinical trial.pt. |  |
|  |  | 212 | randomized controlled trial.pt. |  |
|  |  | 213 | multicenter study.pt. |  |
|  |  | 214 | clinical trial.pt. |  |
|  |  | 215 | exp Clinical Trials as topic/ |  |
|  |  | 216 | (clinical adj trial$).tw. |  |
|  |  | 217 | ((singl$ or doubl$ or treb$ or tripl$) adj (blind$3 or mask$3)).tw. |  |
|  |  | 218 | PLACEBOS/ |  |
|  |  | 219 | placebo$.tw. |  |
|  |  | 220 | randomly allocated.tw. |  |
|  |  | 221 | (allocated adj2 random$).tw. |  |
|  |  | 222 | or/201-221 |  |
|  |  | 223 | Randomization/ |  |
|  |  | 224 | Single blind procedure/ |  |
|  |  | 225 | Double blind procedure/ |  |
|  |  | 226 | Crossover procedure/ |  |
|  |  | 227 | Placebo/ |  |
|  |  | 228 | Randomi?ed controlled trial$.tw. |  |
|  |  | 229 | Rct.tw. |  |
|  |  | 230 | Random allocation.tw. |  |
|  |  | 231 | Allocated randomly.tw. |  |
|  |  | 232 | (allocated adj2 random).tw. |  |
|  |  | 233 | Single blind$.tw. |  |
|  |  | 234 | Double blind$.tw. |  |
|  |  | 235 | ((treble or triple) adj blind$).tw. |  |
|  |  | 236 | Prospective study/ |  |
|  |  | 237 | or/223-236 |  |
|  |  | 238 | 222 or 237 |  |
|  |  | 239 | case report.tw. |  |
|  |  | 240 | historical article/ or Case study/ or Abstract report/ or letter/ |  |
|  |  | 241 | (letter or editorial).pt. |  |
|  |  | 242 | or/239-241 |  |
|  |  | 243 | 238 not 242 |  |
|  |  |  |  |  |
| **MESHED SIGN, Prospective** | #244-263 | 244 | Cohort Studies/ |  |
|  |  | 245 | cohort$.ti |  |
|  |  | 246 | longitudinal.ti |  |
|  |  | 247 | Follow-Up Studies/ |  |
|  |  | 248 | evaluation stud$.ti |  |
|  |  | 249 | Prospective Studies/ |  |
|  |  | 250 | (prospective adj (study or studies)).mp |  |
|  |  | 251 | (observational adj (study or studies)).mp |  |
|  |  | 252 | OR/244-251 |  |
|  |  | 253 | Clinical study/ |  |
|  |  | 254 | Case control study/ |  |
|  |  | 255 | Longitudinal study/ |  |
|  |  | 256 | Prospective study/ |  |
|  |  | 257 | Cohort analysis/ |  |
|  |  | 258 | (Cohort adj (study or studies)).mp |  |
|  |  | 259 | (Case control adj (study or studies)).tw |  |
|  |  | 260 | (follow up adj (study or studies)).tw |  |
|  |  | 261 | (observational adj (study or studies)).tw |  |
|  |  | 262 | Or/253-261 |  |
|  |  | 263 | 252 or 262 |  |
|  |  |  |  |  |
| **MESHED SIGN, Systematic Reviews** | #264-296 | 264 | Meta-Analysis as Topic/ or exp Meta Analysis/ |  |
|  |  | 265 | ((meta analy$) or (meta adj analy$) or metaanalys$).tw |  |
|  |  | 266 | metaanaly$.tw. |  |
|  |  | 267 | Meta-Analysis/ |  |
|  |  | 268 | (systematic adj (review$1 or overview$1)).tw. |  |
|  |  | 269 | exp Review Literature as Topic/ |  |
|  |  | 270 | or/264-269 |  |
|  |  | 271 | cochrane.ab. |  |
|  |  | 272 | embase.ab. |  |
|  |  | 273 | (psychlit or psyclit).ab. |  |
|  |  | 274 | (psychinfo or psycinfo).ab. |  |
|  |  | 275 | (cinahl or cinhal).ab. |  |
|  |  | 276 | science citation index.ab. |  |
|  |  | 277 | bids.ab |  |
|  |  | 278 | or/271-277 |  |
|  |  | 279 | reference list$.ab. |  |
|  |  | 280 | bibliograph$.ab. |  |
|  |  | 281 | hand-search$.ab. |  |
|  |  | 282 | relevant journals.ab. |  |
|  |  | 283 | manual search$.ab. |  |
|  |  | 284 | or/279-283 |  |
|  |  | 285 | selection criteria.ab. |  |
|  |  | 286 | data extraction.ab. |  |
|  |  | 287 | 285 or 286 |  |
|  |  | 288 | Review/ or review.pt |  |
|  |  | 289 | 287 and 288 |  |
|  |  | 290 | (letter or note or editorial or comment or addresses or bibliography or book or book series or chapter or case reports or short survey or conference abstract).pt. |  |
|  |  | 291 | animal/ |  |
|  |  | 292 | human/ |  |
|  |  | 293 | 291 not (291 and 292) |  |
|  |  | 294 | 290 or 293 |  |
|  |  | 295 | 270 or 278 or 284 or 289 |  |
|  |  | 296 | 295 not 294 |  |
|  |  |  |  |  |
| **All populations and interventions, with RCT filter** |  | 297 | 200 AND 243 |  |
|  |  |  |  |  |
| **All populations and interventions, with prospective filter** |  | 298 | 200 AND 263 |  |
|  |  |  |  |  |
| **All populations and interventions, with systematic filter** |  | 299 | 200 AND 296 |  |
|  |  |  |  |  |
| **All populations, interventions, and studies** |  | 300 | OR/297-299 |  |
|  |  | 301 | remove duplicates from 300 |  |

### Table 1B. CINAHL (EBSCO) Search Strategy

| **Term Group** | **Search Number** | **Search Terms** | | |
| --- | --- | --- | --- | --- |
| **Population** | #1-23 | 1 | exp "Attention Deficit and Disruptive Behavior Disorders"/ or exp "Attention Deficit Disorder with Hyperactivity"/ |  |
|  |  | 2 | attention deficit hyperactivity disorder.mp |  |
|  |  | 3 | attention deficit disorder$.mp |  |
|  |  | 4 | attention-deficit hyperactivity disorder.mp |  |
|  |  | 5 | adhd.mp |  |
|  |  | 6 | ad hd.mp |  |
|  |  | 7 | addh.mp |  |
|  |  | 8 | hyperkinetic disorder.mp |  |
|  |  | 9 | (attention deficit AND disruptive behavior$).mp |  |
|  |  | 10 | hyperactiv$.ti |  |
|  |  | 11 | disruptiv$.ti |  |
|  |  | 12 | impulsiv$.ti |  |
|  |  | 13 | inattentiv$.ti |  |
|  |  | 14 | inattention$.ti |  |
|  |  | 15 | hyperkin$.ti |  |
|  |  | 16 | hyper kin$.ti |  |
|  |  | 17 | hkd.ti |  |
|  |  | 18 | minimal brain dysfunction.ti |  |
|  |  | 19 | hyperkinetic syndrome.mp |  |
|  |  | 20 | attention deficit$.mp |  |
|  |  | 21 | attention disturbance.mp |  |
|  |  | 22 | disruptive behavior.mp |  |
|  |  | 23 | OR/1-22 |  |
| **Interventions** |  |  |  |  |
| Methylphenidate | #24-59 | 24 | Methylphenidate/ |  |
|  |  | 25 | Ritalin/ |  |
|  |  | 26 | (Ritalin adj (SR OR LA)).mp |  |
|  |  | 27 | ritaline/ |  |
|  |  | 28 | equasym/ |  |
|  |  | 29 | (equasym adj XL).mp |  |
|  |  | 30 | centedrin/ |  |
|  |  | 31 | phenidylate/ |  |
|  |  | 32 | tsentedrin/ |  |
|  |  | 33 | d erythro methyl phenidylacetate.mp |  |
|  |  | 34 | alpha phenyl alpha 2 piperidly acetic acid methyl ester.mp |  |
|  |  | 35 | alpha phenyl 2 piperidineacetic acid methyl ester.mp |  |
|  |  | 36 | d erythro methyl phenidylacetate.mp |  |
|  |  | 37 | d1 erythro methyl phenidylacetate.mp |  |
|  |  | 38 | (metadate OR (medadate adj (ER OR CD))).mp |  |
|  |  | 39 | methylfenidate.mp |  |
|  |  | 40 | methyl phenidate.ti,ab |  |
|  |  | 41 | methylphenidylacetate.ti,ab |  |
|  |  | 42 | methylphenindate.ti,ab |  |
|  |  | 43 | methylphenydate.mp |  |
|  |  | 44 | methyl 2 phenyl 2 piperid 2 ylacetate.mp |  |
|  |  | 45 | phenidylate.mp |  |
|  |  | 46 | phenidyl hydrochloride.mp |  |
|  |  | 47 | (sr 20 or sr20).mp |  |
|  |  | 48 | (Methylin or (methylin adj ER)).mp. |  |
|  |  | 49 | Attenta.mp |  |
|  |  | 50 | Riphenidate.mp |  |
|  |  | 51 | Ritalina.mp |  |
|  |  | 52 | rubifen.mp |  |
|  |  | 53 | tranquilyn.mp |  |
|  |  | 54 | (medikinet OR (medikinet adj XL)).mp |  |
|  |  | 55 | concerta.mp |  |
|  |  | 56 | (Focalin OR (Focalin adj XR)).mp |  |
|  |  | 57 | daytrana.mp |  |
|  |  | 58 | (Quillivant OR (Quillivant adj ER)).mp |  |
|  |  | 59 | OR/24-58 |  |
|  |  |  |  |  |
| Atomoxetine | #60-78 | 60 | Atomoxetin$.mp |  |
|  |  | 61 | Atomoxetine/ |  |
|  |  | 62 | Tomoxetine.mp |  |
|  |  | 63 | ly 139602.mp |  |
|  |  | 64 | ly 139603.mp |  |
|  |  | 65 | ly139602.mp |  |
|  |  | 66 | ly139603.mp |  |
|  |  | 67 | n methyl gamma 2 methylphenoxy phenylpropylamine.mp |  |
|  |  | 68 | n methyl gamma 2 methylphenoxy benzenepropanamine.mp |  |
|  |  | 69 | n methyl 3 2 methylphenoxy 3 phenylpropylamine.mp |  |
|  |  | 70 | n methyl 3 phenyl 3 ortho tolyloxy propylamine.mp |  |
|  |  | 71 | Strattera.mp |  |
|  |  | 72 | Recit.mp |  |
|  |  | 73 | norepinephrine reuptake inhibitor.mp |  |
|  |  | 74 | 83015 26 3.rn,mp. |  |
|  |  | 75 | 82248 59 7.rn,mp. |  |
|  |  | 76 | 82857 39 4.rn,mp. |  |
|  |  | 77 | 82857 40 7.rn,mp. |  |
|  |  | 78 | OR/60-77 |  |
|  |  |  |  |  |
| Dexamphetamine | #79-138 | 79 | dextroamphetamine/ |  |
|  |  | 80 | dexamphetamine.mp |  |
|  |  | 81 | dexamfetamine.mp |  |
|  |  | 82 | d amphetamine.mp |  |
|  |  | 83 | (Dexedrine OR (Dexedrine adj SR) OR (Dexedrine adj Spansules)).mp |  |
|  |  | 84 | (Dextroamphetamine OR (dextroamphetamine adj ER)).mp |  |
|  |  | 85 | dextro amphetamine.mp |  |
|  |  | 86 | afatin.mp |  |
|  |  | 87 | Afettine.mp |  |
|  |  | 88 | Albemap.mp |  |
|  |  | 89 | amfetasul.mp |  |
|  |  | 90 | amitrene.mp |  |
|  |  | 91 | amphedrine.mp |  |
|  |  | 92 | amphex.mp |  |
|  |  | 93 | amsustain.mp |  |
|  |  | 94 | ardex.mp |  |
|  |  | 95 | betafedrina.mp |  |
|  |  | 96 | betaphedrine.mp |  |
|  |  | 97 | biphetamine.mp |  |
|  |  | 98 | carboxyphen.mp |  |
|  |  | 99 | dadex.mp |  |
|  |  | 100 | methylphenethylamin.mp |  |
|  |  | 101 | (d alpha adj2 methylphenethylamin$).mp |  |
|  |  | 102 | d alpha methylphenethylamine sul?ate.mp |  |
|  |  | 103 | Daprisal.mp |  |
|  |  | 104 | d beta phenylisopropylamine.mp |  |
|  |  | 105 | dephadren.mp |  |
|  |  | 106 | dexadrine.mp |  |
|  |  | 107 | Dexaline.mp |  |
|  |  | 108 | dexalme.mp |  |
|  |  | 109 | dexalone.mp |  |
|  |  | 110 | dexamed.mp |  |
|  |  | 111 | dexamphetamine.mp |  |
|  |  | 112 | dexamphethamine.mp |  |
|  |  | 113 | dexamphoid.mp |  |
|  |  | 114 | dexamyl.mp |  |
|  |  | 115 | (dexaspan adj b).mp |  |
|  |  | 116 | dexeamphetanine.mp |  |
|  |  | 117 | dexoval.mp |  |
|  |  | 118 | dextrostat.mp |  |
|  |  | 119 | diocarb.mp |  |
|  |  | 120 | diocurb.mp |  |
|  |  | 121 | Domafate.mp |  |
|  |  | 122 | Domefate.mp |  |
|  |  | 123 | Doxedrine.mp |  |
|  |  | 124 | Dexmethylphenidate.mp |  |
|  |  | 125 | d 1 phenyl 2 aminopropane.mp |  |
|  |  | 126 | dynaphenyl.mp |  |
|  |  | 127 | evrodex.mp |  |
|  |  | 128 | hetamine.mp |  |
|  |  | 129 | nsc 73713.mp |  |
|  |  | 130 | obesedrin.mp |  |
|  |  | 131 | obesonil.mp |  |
|  |  | 132 | phetadex.mp |  |
|  |  | 133 | (simpamina adj d).mp |  |
|  |  | 134 | sympamin.mp |  |
|  |  | 135 | LiquADD.mp |  |
|  |  | 136 | 51 64 9.rn,mp |  |
|  |  | 137 | Attentin.mp |  |
|  |  | 138 | OR/79-137 |  |
|  |  |  |  |  |
| Lisdexamfetamine / Lisdexamphetamine | #139-155 | 139 | Lisdexam*etamine dimes?late.mp. |  |
|  |  | 140 | Lisdexam*etamine.mp |  |
|  |  | 141 | lis-dexam*etamine dimes?late.mp |  |
|  |  | 142 | lisdexam*etamine mes?late.mp |  |
|  |  | 143 | Lisdexamfetamine/ |  |
|  |  | 144 | Lisdexamphetamine/ |  |
|  |  | 145 | Vyvanse.mp |  |
|  |  | 146 | Elvanse.mp |  |
|  |  | 147 | NRP104.mp |  |
|  |  | 148 | NRP 104.mp |  |
|  |  | 149 | SPD 489.mp |  |
|  |  | 150 | SPD489.mp |  |
|  |  | 151 | 608137 32 2.rn,mp |  |
|  |  | 152 | 608137 33 3.rn,mp |  |
|  |  | 153 | 819871 04 0.rn,mp |  |
|  |  | 154 | lysine 1 methyl phenylethylamide.mp |  |
|  |  | 155 | OR/139-154 |  |
|  |  |  |  |  |
| Guanfacine (extended release /immediate release) | #156-174 | 156 | Intuniv.mp |  |
|  |  | 157 | Intuniv/ |  |
|  |  | 158 | guanfacin$.mp |  |
|  |  | 159 | guanfascin$.mp |  |
|  |  | 160 | guarfacin$.mp |  |
|  |  | 161 | guanfacine/ |  |
|  |  | 162 | ((N adj amidino adj #2 OR #2 adj #6 adj dichlorophenyl) AND acetamide).mp |  |
|  |  | 163 | ((N adj aminoiminomethyl #2 adj #6 adj dichloro) AND benzeneacetamide).mp |  |
|  |  | 164 | Lon 798.mp |  |
|  |  | 165 | BS-100-141.mp |  |
|  |  | 166 | BS 100 141.mp |  |
|  |  | 167 | BS100141.mp |  |
|  |  | 168 | SPD 503.mp |  |
|  |  | 169 | SPD503.mp |  |
|  |  | 170 | 29110 47 2.rn,mp |  |
|  |  | 171 | Tenex.mp |  |
|  |  | 172 | Estulic.mp |  |
|  |  | 173 | Dipresan.mp |  |
|  |  | 174 | OR/156-173 |  |
|  |  |  |  |  |
| Clonidine (immediate release) | #175-198 | 175 | clonidine/ |  |
|  |  | 176 | 4205-90-7.rn,mp |  |
|  |  | 177 | alpha-2 adrenergic agonist.mp |  |
|  |  | 178 | adrenergic alpha-2 receptor agonist.mp |  |
|  |  | 179 | ST 155.mp |  |
|  |  | 180 | ST155.mp |  |
|  |  | 181 | M 5041T.mp |  |
|  |  | 182 | M5041T.mp |  |
|  |  | 183 | Catapres.mp |  |
|  |  | 184 | Catapresan.mp |  |
|  |  | 185 | Catapressan.mp |  |
|  |  | 186 | Chlophazolin.mp |  |
|  |  | 187 | Clofelin.mp |  |
|  |  | 188 | Clofenil.mp |  |
|  |  | 189 | Clopheline.mp |  |
|  |  | 190 | Dixarit.mp |  |
|  |  | 191 | Gemiton.mp |  |
|  |  | 192 | Hemiton.mp |  |
|  |  | 193 | Isoglaucon.mp |  |
|  |  | 194 | Klofelin.mp |  |
|  |  | 195 | Klofenil.mp |  |
|  |  | 196 | Jenloga.mp |  |
|  |  | 197 | (Imidazol 2 amine N 2 6 dichlorophenyl 4 5 dihydro).mp |  |
|  |  | 198 | OR/175-197 |  |
|  |  |  |  |  |
| **All interventions** |  | 199 | 59 or 78 or 138 or 155 or 174 or 198 |  |
|  |  |  |  |  |
| **All populations and interventions** |  | 200 | 23 AND 199 |  |
|  |  |  |  |  |
| **MESHED SIGN, RCT** | #201-243 | 201 | Randomized Controlled Trials as Topic/ |  |
|  |  | 202 | randomized controlled trial/ |  |
|  |  | 203 | Random Allocation/ |  |
|  |  | 204 | Double Blind Method/ |  |
|  |  | 205 | Single Blind Method/ |  |
|  |  | 206 | clinical trial/ |  |
|  |  | 207 | clinical trial, phase i.pt. |  |
|  |  | 208 | clinical trial, phase ii.pt. |  |
|  |  | 209 | clinical trial, phase iii.pt. |  |
|  |  | 210 | clinical trial, phase iv.pt. |  |
|  |  | 211 | controlled clinical trial.pt. |  |
|  |  | 212 | randomized controlled trial.pt. |  |
|  |  | 213 | multicenter study.pt. |  |
|  |  | 214 | clinical trial.pt. |  |
|  |  | 215 | exp Clinical Trials as topic/ |  |
|  |  | 216 | (clinical adj trial$).tw. |  |
|  |  | 217 | ((singl$ or doubl$ or treb$ or tripl$) adj (blind$3 or mask$3)).tw. |  |
|  |  | 218 | PLACEBOS/ |  |
|  |  | 219 | placebo$.tw. |  |
|  |  | 220 | randomly allocated.tw. |  |
|  |  | 221 | (allocated adj2 random$).tw. |  |
|  |  | 222 | or/201-221 |  |
|  |  | 223 | Randomization/ |  |
|  |  | 224 | Single blind procedure/ |  |
|  |  | 225 | Double blind procedure/ |  |
|  |  | 226 | Crossover procedure/ |  |
|  |  | 227 | Placebo/ |  |
|  |  | 228 | Randomi?ed controlled trial$.tw. |  |
|  |  | 229 | Rct.tw. |  |
|  |  | 230 | Random allocation.tw. |  |
|  |  | 231 | Allocated randomly.tw. |  |
|  |  | 232 | (allocated adj2 random).tw. |  |
|  |  | 233 | Single blind$.tw. |  |
|  |  | 234 | Double blind$.tw. |  |
|  |  | 235 | ((treble or triple) adj blind$).tw. |  |
|  |  | 236 | Prospective study/ |  |
|  |  | 237 | or/223-236 |  |
|  |  | 238 | 222 or 237 |  |
|  |  | 239 | case report.tw. |  |
|  |  | 240 | historical article/ or Case study/ or Abstract report/ or letter/ |  |
|  |  | 241 | (letter or editorial).pt. |  |
|  |  | 242 | or/239-241 |  |
|  |  | 243 | 238 not 242 |  |
|  |  |  |  |  |
| **MESHED SIGN, Prospective** | #244-263 | 244 | Cohort Studies/ |  |
|  |  | 245 | cohort$.ti |  |
|  |  | 246 | longitudinal.ti |  |
|  |  | 247 | Follow-Up Studies/ |  |
|  |  | 248 | evaluation stud$.ti |  |
|  |  | 249 | Prospective Studies/ |  |
|  |  | 250 | (prospective adj (study or studies)).mp |  |
|  |  | 251 | (observational adj (study or studies)).mp |  |
|  |  | 252 | OR/244-251 |  |
|  |  | 253 | Clinical study/ |  |
|  |  | 254 | Case control study/ |  |
|  |  | 255 | Longitudinal study/ |  |
|  |  | 256 | Prospective study/ |  |
|  |  | 257 | Cohort analysis/ |  |
|  |  | 258 | (Cohort adj (study or studies)).mp |  |
|  |  | 259 | (Case control adj (study or studies)).tw |  |
|  |  | 260 | (follow up adj (study or studies)).tw |  |
|  |  | 261 | (observational adj (study or studies)).tw |  |
|  |  | 262 | Or/253-261 |  |
|  |  | 263 | 252 or 262 |  |
|  |  |  |  |  |
| **MESHED SIGN, Systematic Reviews** | #264-296 | 264 | Meta-Analysis as Topic/ or exp Meta Analysis/ |  |
|  |  | 265 | ((meta analy$) or (meta adj analy$) or metaanalys$).tw |  |
|  |  | 266 | metaanaly$.tw. |  |
|  |  | 267 | Meta-Analysis/ |  |
|  |  | 268 | (systematic adj (review$1 or overview$1)).tw. |  |
|  |  | 269 | exp Review Literature as Topic/ |  |
|  |  | 270 | or/264-269 |  |
|  |  | 271 | cochrane.ab. |  |
|  |  | 272 | embase.ab. |  |
|  |  | 273 | (psychlit or psyclit).ab. |  |
|  |  | 274 | (psychinfo or psycinfo).ab. |  |
|  |  | 275 | (cinahl or cinhal).ab. |  |
|  |  | 276 | science citation index.ab. |  |
|  |  | 277 | bids.ab |  |
|  |  | 278 | or/271-277 |  |
|  |  | 279 | reference list$.ab. |  |
|  |  | 280 | bibliograph$.ab. |  |
|  |  | 281 | hand-search$.ab. |  |
|  |  | 282 | relevant journals.ab. |  |
|  |  | 283 | manual search$.ab. |  |
|  |  | 284 | or/279-283 |  |
|  |  | 285 | selection criteria.ab. |  |
|  |  | 286 | data extraction.ab. |  |
|  |  | 287 | 285 or 286 |  |
|  |  | 288 | Review/ or review.pt |  |
|  |  | 289 | 287 and 288 |  |
|  |  | 290 | (letter or note or editorial or comment or addresses or bibliography or book or book series or chapter or case reports or short survey or conference abstract).pt. |  |
|  |  | 291 | animal/ |  |
|  |  | 292 | human/ |  |
|  |  | 293 | 291 not (291 and 292) |  |
|  |  | 294 | 290 or 293 |  |
|  |  | 295 | 270 or 278 or 284 or 289 |  |
|  |  | 296 | 295 not 294 |  |
|  |  |  |  |  |
| **All populations and interventions, with RCT filter** |  | 297 | 200 AND 243 |  |
|  |  |  |  |  |
| **All populations and interventions, with prospective filter** |  | 298 | 200 AND 263 |  |
|  |  |  |  |  |
| **All populations and interventions, with systematic filter** |  | 299 | 200 AND 296 |  |
|  |  |  |  |  |
| **All populations, interventions, and studies** |  | 300 | OR/297-299 |  |
|  |  | 301 | remove duplicates from 300 |  |

### Table 1C. Science Citation Index (SCI) [Web of Knowledge] Search Strategy

| **Term Group** | **Search Number** | **Search Terms** | | |
| --- | --- | --- | --- | --- |
| **Population** | #1 | 1 | TS="Attention Deficit and Disruptive Behavior Disorders" or TS="attention deficit hyperactivity disorder*" or TS="attention deficit disorder*" or TS="attention-deficit hyperactivity disorder*" or TS=adhd* or TS="ad hd" or TS=addh or TS="hyperkinetic disorder*" or TS=("attention deficit” AND “disruptive behavior*") or TI=hyperactiv* or TI=disruptiv* or TI=impulsiv* or TI=inattentiv* or TI=Inattention* or TI=Hyperkin* or TI="hyper kin*" or TI=hkd or TI="minimal brain dysfunction" or TS="hyperkinetic syndrome" or TS="Attention deficit*" or TS="attention disturbance" or TS="disruptive behavior" |  |
|  |  |  |  |  |
| **Intervention** |  |  |  |  |
| Methylphenidate | #2 | 2 | TS=Methylphenidate or TS=Ritalin or TS=("Ritalin SR" OR "Ritalin LA") or TS=ritaline or TS=equasym or TS="equasym XL" or TS=centedrin or TS=phenidylate or TS=tsentedrin or TS="d erythro methyl phenidylacetate" or TS="alpha phenyl alpha 2 piperidly acetic acid methyl ester" or TS="alpha phenyl 2 piperidineacetic acid methyl ester" or TS="d erythro methyl phenidylacetate" or TS="d1 erythro methyl phenidylacetate" or TS=(metadate OR "medadate ER" OR "metadate CD") or TS=methylfenidate or TS="methyl phenidate" or TS=methylphenidylacetate or TS=methylphenindate or TS=methylphenydate or TS="methyl 2 phenyl 2 piperid 2 ylacetate" or TS=phenidylate or TS="phenidyl hydrochloride" or TS="sr 20" or TS=(Methylin or "methylin ER") or TS=Attenta or TS=Riphenidate or TS=Ritalina or TS=rubifen or TS=tranquilyn or TS=(medikinet OR "medikinet XL") or TS=(Focalin OR "Focalin XR" OR concerta) or TS=(Daytrana OR Quillivant OR "Quillivant ER") |  |
|  |  |  |  |  |
| Atomoxetine | #3 | 3 | TS=atomoxetin* or S=tomoxetine or TS="ly 139602" or TS="ly 139603" or TS="ly139602" or TS="ly139603" or TS="n methyl gamma 2 methylphenoxy phenylpropylamine" or TS="n methyl gamma 2 methylphenoxy benzenepropanamine" or TS="n methyl 3 2 methylphenoxy 3 phenylpropylamine" or TS="n methyl 3 phenyl 3 ortho tolyloxy propylamine" or TS=Strattera or TS=Recit or TS="norepinephrine reuptake inhibitor" or TS = "83015 26 3" |  |
|  |  |  |  |  |
| Dexamphetamine | #4 | 4 | TS=dextroamphetamine or TS=dexamphetamine or TS=dexamfetamine or TS="d amphetamine" or TS=(Dexedrine OR "Dexedrine SR" OR "Dexedrine Spansules") or TS=(Dextroamphetamine OR "dextroamphetamine ER") or TS="dextro amphetamine" or TS=afatin or TS=Afettine or TS=Albemap or TS=amfetasul or TS=amitrene or TS=amphedrine or TS=amphex or TS=amsustain or TS=ardex or TS=betafedrina or TS=betaphedrine or TS=biphetamine or TS=carboxyphen or TS=dadex or TS=methylphenethylamin or TS="d alpha methylphenethylamine sul*ate" or TS=Daprisal or TS="d beta phenylisopropylamine" or TS=dephadren or TS=dexadrine or TS=Dexaline or TS=dexalme or TS=dexalone or TS=dexamed or TS=dexamphetamine or TS=dexamphethamine or TS=dexamphoid or TS=dexamyl or TS="dexaspan b" or TS=dexeamphetanine or TS=dexoval or TS=dextrostat or TS=diocarb or TS=diocurb or TS=Domafate or TS=Domefate or TS=Doxedrine or TS=Dexmethylphenidate or TS="d 1 phenyl 2 aminopropane" or TS=dynaphenyl or TS=evrodex or TS=hetamine or TS="nsc 73713" or TS=obesedrin or TS=obesonil or TS=phetadex or TS="simpamina d" or TS=sympamin or TS="51 64 9" or TS=LiquADD or TS=Attentin |  |
|  |  |  |  |  |
| Lisdexamfetamine / Lisdexamphetamine | #5 | 5 | TS="Lisdexam*etamine dimes?late" or TS=Lisdexam*etamine or TS="lis-dexam*etamine dimes?late" or TS="lisdexam*etamine mes?late" or TS="lysine 1 methyl phenylethylamide" or TS=Vyvanse or TS=Elvanse or TS=NRP104 or TS="NRP 104" or TS="SPD 489" or TS=SPD489 or TS="608137 32 2" or TS="608137 33 3" or TS="819871040" |  |
|  |  |  |  |  |
| Guanfacine (extended release /immediate release) | #6 | 6 | TS=Intuniv or TS=guanfacine* or TS=guanfascin* or TS=guarfacin* or TS=guanfacine or TS=((N NEXT amidino NEXT 2 OR 2 NEXT 6 NEXT dichlorophenyl) AND acetamide) or TS=((N NEXT aminoiminomethyl 2 NEXT 6 NEXT dichloro) AND benzeneacetamide) or TS="Lon 798" or TS="BS-100-141" or TS="BS 100 141" or TS=BS100141 or TS="SPD 503" or TS=SPD503 or TS="29110 47 2" or TS=Tenex or TS=Estulic or TS=Dipresan |  |
|  |  |  |  |  |
| Clonidine (immediate release) | #7 | 7 | TS=clonidine or TS=4205-90-7 or TS="alpha-2 adrenergic agonist" or TS="adrenergic alpha-2 receptor agonist" or TS="ST 155" or TS=ST155 or TS="M 5041T" or TS=M5041T or TS=Catapres or TS=Catapresan or TS=Catapressan or TS=Chlophazolin or TS=Clofelin or TS=Clofenil or TS=Clopheline or TS=Dixarit or TS=Gemiton or TS=Hemiton or TS=Isoglaucon or TS=Klofelin or TS=Klofenil or TS=Jenloga or TS=(Imidazol 2 amine N 2 6 dichlorophenyl 4 5 dihydro) |  |
|  |  |  |  |  |
| **All interventions** | #8 | 8 | #2 OR #3 OR #4 OR #5 OR #6 OR #7 |  |
|  |  |  |  |  |
| **All populations and interventions** | #9 | 9 | #1 AND #8 |  |
|  |  |  |  |  |
| **Publication types** |  |  |  |  |
| Filter, RCT | #10-12 | 10 | TS="clinical trial" or S="randomized controlled trial" or TS="randomization" or TS="single blind procedure" or TS="double blind procedure" or TS="crossover procedure" or TS="placebo" or TS="randomi?ed controlled trial*" or TS=Rct or TS="random allocation" or TS="randomly allocated" or TS="allocated randomly" or TS=(allocated SAME random) or TS="single blind*" or TS="double blind*" or TS=((treble or triple) NEXT (blind*)) or TS=placebo* or TS="prospective study" |  |
|  |  | 11 | TS="case study" or TS="case report" or TS=("abstract report" or letter) |  |
|  |  | 12 | #10 NOT #11 |  |
|  |  |  |  |  |
| Filter, Systematic Reviews | #13-15 | 13 | TS="meta analysis" or TS=((meta NEXT analy*) or metaanalys*) or TS=(systematic NEXT/1 (review* or overview*)) or TS=cochrane or TS=embase or TS=(psychlit or psyclit) or TS=(psychinfo or psycinfo) or TS=(cinahl or cinhal) or TS=bids or TS="reference lists" or TS=bibliograph* or TS=hand-search* or TS="manual search*" or TS="relevant journals" or TS=review |  |
|  |  | 14 | TS=(("letter" OR "editorial") OR ("animal" not ("animal and human"))) |  |
|  |  | 15 | #13 NOT #14 |  |
|  |  |  |  |  |
| Filter, Prospective Studies | #16 | 16 | TS="clinical study" or TS="case control study" or TS="longitudinal study" or TS="prospective study" or TS="cohort analysis" or TS=(cohort NEXT/1 (study or studies)) or TS=(case control NEXT/1 (study or studies)) or TS=("follow up" NEXT/1 (study or studies)) or TS=(observational NEXT/1 (study or studies)) |  |
|  |  |  |  |  |
| **All studies, populations, and interventions** | #17-20 | 17 | #9 AND #12 |  |
|  |  | 18 | #9 AND #13 |  |
|  |  | 19 | #9 AND #14 |  |
|  |  | 20 | #17 OR #18 OR #19 |  |

# Inclusion and Exclusion Criteria

The systematic review will be conducted in two levels. During Level I, titles and abstracts will be reviewed for clear exclusion criteria. The inclusion and exclusion criteria for the Level I screening are documented in **Table 2**. Selected literature will include RCTs that report any efficacy outcomes of interest listed in the inclusion criteria. If a study is excluded, the reason for exclusion will be recorded.

For the abstracts that pass Level I screening or those with uncertainties based on the Level I screening, the corresponding full articles will be retrieved for further review during the Level II screening. The same inclusion and exclusion criteria as in the Level I screening will be applied in the Level II screening. For any excluded studies, the reason for exclusion of individual studies will also be recorded. Multiple publications of the same patient population and reporting the same outcomes (i.e., one is primary publication and the other is a review article) will be identified as “kin” studies and linked and reviewed to avoid duplication. The primary or “parent” study will be identified and will be used as the paper from which the majority of the data is extracted.

In addition, review articles or meta-analysis articles will be reviewed to identify additional studies (i.e., the snowballing technique) or confirm the identified studies that will be selected for data extraction in follow-up analyses. Finally, a PRISMA diagram will be produced to describe the study selection process, reasons for exclusion per level of screening, and the list of articles selected for inclusion.

To ensure the accuracy of selected studies at each level of screening and the quality assessment, two researchers will conduct these activities independently. If there is uncertainty regarding the relevance of the study or disagreement on the quality assessment, the researcher will consult a third reviewer to reach a consensus.

### Table 2: Inclusion and exclusion criteria of the systematic search

| Inclusion criteria | **Population:** Children and adolescents with ADHD (6-17 years old^[[4]](#footnote-5)^), or ADHD trials in which population outcomes are reported separately for children or adolescents.  **Interventions**:  Interventions comprised of the following monotherapies, will be included:   - Methylphenidate (MPH) monotherapy   - Methylphenidate extended release (OROS-MPH / MPH intermediate release)   - Methylphenidate immediate release (MPH-IR) - Atomoxetine (ATX) monotherapy - Dexamfetamine (d-AMPH) monotherapy - Lisdexamfetamine / Lisdexamphetamine (LDX) monotherapy - Guanfacine monotherapy   - Guanfacine extended release (GXR)   - Guanfacine immediate release (GIR) - Clonidine immediate release (CIR) monotherapy   **Study design:**  Trials with the following study design will be considered:  Phase 2 or Phase 3 randomized controlled trials with comparator arms (RCTs) of study length less than or equal to 16 weeks, or longer studies that present interim results at or before 16 weeks, are eligible for inclusion; cross-over RCTs will be included if data are presented at cross-over.  **Outcomes:**  The trial reports at least one efficacy or safety outcome in the following categories:   - ADHD symptom measures (ADHD-RS-IV or CGI-I) - Discontinuation due to any reasons or due to adverse events   **Publication type:**  The following types of publications are eligible for inclusion:   - Published literature and conference abstracts that meet study design criteria stated above - Abstracts that meet the inclusion criteria will be included if an associated full-text published paper(s) cannot be identified   **Language:** English (non-English publications will be noted but will not be included in the data extraction or MTC)  **Sample size:** Greater than 25 ADHD patients |
| --- | --- |

| Exclusion criteria | **Study population:**   - *Only* adult ADHD patients OR - Includes adults and children or adolescents with ADHD, but the outcome for children or adolescents was not reported separately - All patients have ADHD plus a comorbidity   **Interventions:** Does *not* include any drug treatments of interest  **Study design:**   - Prospective non-RCT studies such as long-term open-label follow-up studies, prospective observational studies (e.g., Phase 4 trials), and non-randomized trials - Retrospective studies - Non-human or in vitro studies - Phase I studies and preclinical studies - Case reports, commentaries, letters, consensus reports - RCTs longer than 16 weeks that do not report interim results at or before 16 weeks   **Outcomes:** Does *not* include sufficient data on the efficacy or discontinuation outcomes listed above in the inclusion criteria  **Publication type:**   - Unpublished and grey literature^[[5]](#footnote-6)^ - Duplicate publication of the same trial - Case reports - Commentaries and letters - Recommendations/guidelines - Non-systematic reviews - Systematic reviews and meta-analyses will not be directly excluded, but will be used to identify or confirm selected studies   **Language:** Non-English publications  **Sample size:** Less than or equal to 25 ADHD patients, or has more than 25 patients but only 25 or less have ADHD |
| --- | --- |

1. The format and language of this study protocol are consistent with the “Specification for manufacturer/sponsor submission of evidence” from NICE. [↑](#footnote-ref-2)
2. The NICE specification for manufacturer/sponsor submission of evidence can be found at: www.nice.org.uk/aboutnice/howwework/devnicetech/SpecificationForManufacturerSponsorSubmissionOfEvidence.jsp?domedia=1&mid=97E2DC44-19B9-E0B5-D4F73F1DB153AA86 [↑](#footnote-ref-3)
3. Scottish Intercollegiate Guidelines Network, Healthcare Improvement Scotland. Search Filters. August 27, 2015. <http://www.sign.ac.uk/methodology/filters.html>. Accessed on: September 21, 2016. [↑](#footnote-ref-4)
4. ADHD trials including individuals between 17 and 18 years old will be included as well. [↑](#footnote-ref-5)
5. Grey literature includes informally published written material (such as reports) that may not be published in peer-reviewed journals or other commercial sources. [↑](#footnote-ref-6)
