# Supplementary material for: Comparative efficacy and safety of attention-deficit/hyperactivity disorder pharmacotherapies, including guanfacine extended release: a mixed treatment comparison
Source: Eur Child Adolesc Psychiatry. 2017 Mar 3;26(8):875–97. doi: 10.1007/s00787-017-0962-6 (PMC5532417; doi:10.1007/s00787-017-0962-6)
Supplement: Supplementary file 2 — Supplementary material 2 (DOCX 50 kb) [file 787_2017_962_MOESM2_ESM.docx]

**Comparative efficacy and safety of attention-deficit/hyperactivity disorder pharmacotherapies, including guanfacine extended release: a mixed treatment comparison**

Alain Joseph^1^, Rajeev Ayyagari^2^, Meng Xie^2^*, Sean Cai^3^*, Jipan Xie^3^, Michael Huss^4^, Vanja Sikirica^5^*

^1^Shire, Zug, Switzerland; ^2^Analysis Group, Inc., Boston, MA, USA; ^3^Analysis Group, Inc., New York, NY, USA; ^4^University Medicine, Dept. of Child and Adolescent Psychiatry, Mainz, Germany; ^5^Shire, Wayne, PA, USA

*Affiliation at time of study

**Table: Baseline characteristics of the included studies**

| **Author (Year)** | **Arms** | **ODD (%)** | **Randomized sample size** | **Full analysis sample size** | **Mean age** | **N (%) female** | **Baseline  ADHD-RS-IV score** |
| --- | --- | --- | --- | --- | --- | --- | --- |
| Dittmann (2013) | LDX | 10.2 | 133 | 128 | 10.9 | 34 (26.6) | 42.6 |
|  | ATX | 9.7 | 134 | 134 | 10.4 | 31 (23.1) | 41.9 |
| Hervas (2014) | GXR | 14.9 | 115 | 114 | 10.9 | 38 (33.3) | 43.1 |
|  | ATX | 8.9 | 112 | 112 | 10.5 | 25 (22.3) | 43.7 |
|  | PBO | 12.6 | 111 | 111 | 11.0 | 25 (22.5) | 43.2 |
| Biederman (2003) | MPH-ER | NA | 66 | 65 | 9.1 | 13 (20.0) | NA |
|  | PBO | NA | 71 | 71 | 8.8 | 19 (26.8) | NA |
| Biederman (2007) | LDX (30 mg) | NA | 71 | 71 | 9.0 | 18 (25.4) | NA |
|  | LDX (50 mg) | NA | 74 | 74 | 8.9 | 28 (37.8) | NA |
|  | LDX (70 mg) | NA | 73 | 73 | 8.7 | 21 (28.8) | NA |
|  | PBO | NA | 72 | 72 | 9.4 | 22 (30.6) | NA |
| Biederman (2008) | GXR (2 mg) | NA | 87 | 87 | 10.6 | 20 (23.0) | 36.6 |
|  | GXR (3 mg) | NA | 86 | 86 | 10.8 | 17 (19.8) | 37.2 |
|  | GXR (4 mg) | NA | 86 | 86 | 10.1 | 29 (33.7) | 38.5 |
|  | PBO | NA | 86 | 86 | 10.6 | 22 (25.6) | 38.1 |
| Block (2009) | ATX (AM+PM) | 30.2 | 195 | 195 | 8.9 | 55 (28.2) | NA |
|  | ATX (AM) | 34.3 | 102 | 102 | 8.8 | 33 (32.5) | NA |
|  | ATX (PM) | 25.8 | 93 | 93 | 9.1 | 22 (23.6) | NA |
|  | PBO | 34.4 | 93 | 93 | 8.9 | 24 (25.8) | NA |
| Coghill (2013) | LDX | 7.2 | 113 | 111 | 10.9 | 24 (21.6) | 41.0 |
|  | MPH-OROS | 9.0 | 112 | 111 | 10.9 | 21 (18.9) | 40.4 |
|  | PBO | 7.3 | 111 | 110 | 11.0 | 19 (17.3) | 41.2 |
| Dittmann (2011) | ATX (fast titration) | 73.3 | 60 | 60 | 11.1 | 9 (15.0) | NA |
|  | ATX (slow titration) | 73.8 | 61 | 61 | 10.8 | 8 (13.1) | NA |
|  | PBO | 76.3 | 59 | 59 | 11.1 | 11 (18.6) | NA |
| Findling (2006) | MPH-IR | NA | 133 | 133 | 9.5 | 28 (21.1) | NA |
|  | MPH-ER | NA | 139 | 139 | 9.5 | 27 (19.4) | NA |
|  | PBO | NA | 46 | 46 | 9.5 | 11 (23.9) | NA |
| Findling (2008) | MTS (methylphenidate transdermal system patch) | NA | 100 | 100 | 8.9 | 40 (40.0) | 43.0 |
|  | MPH-OROS | NA | 94 | 94 | 8.8 | 32 (34.0) | 43.8 |
|  | PBO | NA | 88 | 88 | 8.5 | 23 (26.1) | 41.9 |
| Findling (2011) | LDX (30 mg) | NA | 78 | 78 | NA | NA | 38.3 |
|  | LDX (50 mg) | NA | 77 | 77 | NA | NA | 37.3 |
|  | LDX (70 mg) | NA | 78 | 78 | NA | NA | 37.0 |
|  | PBO | NA | 77 | 77 | NA | NA | 38.5 |
| Garg (2014) | MPH-IR | 45.5 | 41 | 33 | 8.5 | 6 (18) | NA |
|  | ATX | 61.1 | 43 | 36 | 8.7 | 7 (19) | NA |
| Gau (2006) | MPH-OROS | NA | 32 | 32 | 10.9 | NA | NA |
|  | MPH-IR | NA | 32 | 32 | 10.1 | NA | NA |
| Gau (2007) | ATX | 19.4 | 72 | 72 | 9.1 | 7 (9.7) | 36.7 |
|  | PBO | 8.8 | 34 | 34 | 9.5 | 5 (14.7) | 37.1 |
| Greenhill (2002) | MPH-MR | NA | 158 | 155 | 9.0 | 27 (17.0) | NA |
|  | PBO | NA | 163 | 159 | 9.0 | 30 (19.0) | NA |
| Ialongo (1994) | MPH-IR (0.4 mg/kg) | 12 (in entire study, numbers not reported by arm) | 16 | 16 | NA | NA | NA |
|  | MPH-IR (0.8 mg/kg) |  | 16 | 16 | NA | NA | NA |
|  | Placebo |  | 16 | 16 | NA | NA | NA |
| Kelsey (2004) | ATX | 37.6 | 133 | 133 | 9.5 | 39 (29.3) | 42.1 |
|  | PBO | 29.7 | 64 | 64 | 9.4 | 19 (29.7) | 42.3 |
| Kemner (2005) | MPH-OROS | NA | 850 | 850 | 8.8 | 219 (25.8) | 39.9 |
|  | ATX | NA | 473 | 473 | 9.2 | 121 (25.6) | 38.6 |
| Kollins (2011) | GXR | NA | 121 | 121 | 12.6 | 41 (33.9) | NA |
|  | PBO | NA | 57 | 57 | 12.8 | 13 (22.8) | NA |
| Lopez (2003) | Ritalin (MPH-ER) | NA | 36 | 36 | 9 | 7 (19.4) | NA |
|  | Concerta (MPH-ER) (18 mg) | NA |  |  |  |  | NA |
|  | Concerta (MPH-ER) (36 mg) | NA |  |  |  |  | NA |
|  | Placebo | NA |  |  |  |  | NA |
| Martenyi (2010) | ATX | 1.4 | 72 | 72 | 9.9 | 9 (12.5) | 38.1 |
|  | PBO | 3.0 | 33 | 33 | 9.6 | 6 (18.2) | 37.0 |
| Michelson (2001) | ATX (0.5 mg) | 47.7 | 44 | 44 | 11.3 | 13 (29.5) | 40.2 |
|  | ATX (1.2 mg) | 29.8 | 84 | 84 | 11.5 | 24 (28.6) | 39.2 |
|  | ATX (1.8 mg) | 42.4 | 85 | 85 | 11.1 | 24 (28.2) | 39.7 |
|  | PBO | 36.7 | 84 | 84 | 10.9 | 24 (28.6) | 38.3 |
| Michelson (2002) | ATX | 18.8 | 85 | 85 | 10.1 | 25 (29.4) | 37.6 |
|  | PBO | 21.2 | 86 | 85 | 10.5 | 25 (29.4) | 36.7 |
| Montoya (2009) | ATX | 28.3 | 100 | 100 | 10.3 | 21 (21.0) | 39.1 |
|  | PBO | 20.0 | 51 | 51 | 10.3 | 10 (19.6) | 39.5 |
| Newcorn (2008) | ATX | 39.0 | 222 | 222 | 10.3 | 50 (23.0) | 40.9 |
|  | MPH-OROS | 36.0 | 220 | 220 | 10.2 | 64 (29.0) | 40.0 |
|  | PBO | 35.0 | 74 | 74 | 10.1 | 19 (26.0) | 41.7 |
| Newcorn (2013) | GXR (AM+PM) | NA | 227 | 221 | 9.2 | 71 (32.1) | 41.7 |
|  | GXR (AM) | NA | 113 | 107 | 9.1 | 35 (32.7) | 41.7 |
|  | GXR (PM) | NA | 114 | 114 | 9.3 | 36 (31.6) | 41.6 |
|  | PBO | NA | 113 | 112 | 8.9 | 27 (24.1) | 42.9 |
| Palumbo (2008) | MPH | 44.8 | 29 | 29 | 9.4 | (17.2) | NA |
|  | Clonidine | 43.3 | 31 | 31 | 9.4 | (12.9) | NA |
|  | Combination (clonidine + MPH) | 50.0 | 32 | 32 | 10.0 | (25.0) | NA |
|  | PBO | 50.0 | 30 | 30 | 9.0 | (23.3) | NA |
| Pliszka (2000) | Adderall | 12.0 | 20 | 20 | 8.6 | NA | NA |
|  | MPH | 14.0 | 20 | 20 | 8.1 | NA | NA |
|  | PBO | 10.0 | 18 | 18 | 7.8 | NA | NA |
| Sallee (2009) | GXR (1 mg) | NA | 62 | NA | NA | NA | NA |
|  | GXR (2 mg) | NA | 65 | NA | NA | NA | NA |
|  | GXR (3 mg) | NA | 65 | NA | NA | NA | NA |
|  | GXR (4 mg) | NA | 66 | NA | NA | NA | NA |
|  | PBO | NA | 66 | NA | NA | NA | NA |
| Shang (2015) | OROS MPH | NA | 80 | 80 | 9.6 | 10 (12.5) | Inattention: 22.58 Hyperactivity: 15.94 |
|  | ATX | NA | 80 | 80 | 9.9 | 10 (12.5) | Inattention: 22.62 Hyperactivity: 17.03 |
| Spencer (2002a) | ATX | NA | 73 | NA | NA | NA | 41.2 |
|  | PBO | NA | 71 | NA | NA | NA | 41.4 |
| Spencer (2002b) | ATX | NA | 56 | NA | NA | NA | 37.8 |
|  | PBO | NA | 23 | NA | NA | NA | 37.6 |
| Steele (2006) | MPH-OROS | 43.1 | 73 | 72 | 9.0 | 11 (15.3) | NA |
|  | MPH-IR | 38.4 | 74 | 73 | 9.1 | 13 (17.8) | NA |
| Su (2016) | OROS MPH | 35.3 | 130 | 119 | 9.5 | 20 (16.8) | 32.5 |
|  | ATX | 25.4 | 132 | 118 | 9.5 | 20 (16.9) | 31.5 |
| Takahashi (2009) | ATX (0.5 mg) | 13.5 | 62 | 62 | 10.3 | 10 (16.1) | NA |
|  | ATX (1.2 mg) |  | 60 | 60 | 10.6 | 8 (13.3) | NA |
|  | ATX (1.8 mg) |  | 61 | 61 | 10.5 | 8 (13.1) | NA |
|  | PBO |  | 62 | 62 | 10.8 | 10 (16.1) | NA |
| Wang (2007) | ATX | NA | 164 | 164 | 9.4 | 28 (17.7) | 38.6 |
|  | MPH | NA | 166 | 166 | 9.9 | 32 (19.3) | 37.4 |
| Wehmeier (2012) | ATX | 31.7 | 63 | 63 | 9.1 | 16 (25.4) | 37.3 |
|  | PBO | 30.6 | 62 | 62 | 8.9 | 12 (19.4) | 36.7 |
| Weiss (2005) | ATX | 32.7 | 101 | 101 | 9.9 | 18 (17.8) | NA |
|  | PBO | 34.6 | 52 | 52 | 9.9 | 12 (23.1) | NA |
| Wigal (2004) | MPH(d) | NA | 44 | 44 | 10.0 | 3 (6.8) | NA |
|  | MPH(d,l) | NA | 46 | 46 | 9.8 | 6 (13.0) | NA |
|  | PBO | NA | 42 | 42 | 9.6 | 7 (16.7) | NA |
| Wilens (2006) | MPH-OROS | NA | 87 | 87 | 14.8 | 23 (26.4) | 31.6 |
|  | PBO | NA | 90 | 90 | 14.5 | 12 (13.3) | 31.0 |
| Wilens (2011) | ATX | NA | 50 | 49 | 8.7 | 15 (31.0) | 42.9 |
|  | PBO | NA | 47 | 46 | 8.6 | 18 (39.0) | 42.9 |
| Wilens (2015) | GXR | 12.7 | 157 | 157 | 14.5 | 54 (34.4) | NA |
|  | Placebo | 10.3 | 157 | 157 | 14.6 | 56 (36.1) | NA |
| Wolraich (2001) | MPH-OROS | 36.8 | 95 | 95 | 8.8 | 21 (22.1) | NA |

ADHD-RS-IV, Attention-Deficit/Hyperactivity Disorder Rating Scale Version IV; ATX, atomoxetine; ER, extended release; GXR, guanfacine extended release; IR, immediate release; LDX, lisdexamfetamine dimesylate; MPH, methylphenidate; NA, not applicable; ODD, oppositional defiant disorder; OROS, osmotic-release oral system; PBO, placebo.
